# Transcriptome analysis of a dog model of congestive heart failure shows that collagen-related 2-oxoglutarate-dependent dioxygenases contribute to heart failure

**DOI:** 10.1038/s41598-022-26717-7

**Published:** 2022-12-29

**Authors:** Takahiro Isono, Takehiro Matsumoto, Masafumi Suzaki, Shigehisa Kubota, Susumu Kageyama, Akihiro Kawauchi, Atuyuki Wada

**Affiliations:** 1 Department of Urology, Shiga University of Medical Science, Otsu, Shiga 520-2192 Japan; 2 Central Research Laboratory, Shiga University of Medical Science, Otsu, Shiga 520-2192 Japan; 3 Center of Cardiovascular Disease and Heart Failure, Omi Medical Center, Kusatsu, Shiga 525-8585 Japan

**Keywords:** Molecular biology, Cardiology

## Abstract

Fibrosis is an important pathological mechanism in heart failure (HF) and is associated with poor prognosis. We analyzed fibrosis in HF patients using transcriptomic data. Genes differentially expressed between normal control and congestive HF (CHF) dogs included *P3H1*, *P3H2*, *P3H4*, *P4HA2*, *PLOD1* and *PLOD3*, which belong to the 2-oxoglutarate-dependent dioxygenases (2OGD) superfamily that stabilizes collagen during fibrosis. Quantitative polymerase chain reaction analysis demonstrated 2OGD gene expression was increased in CHF samples compared with normal left ventricle (LV) samples. 2OGD gene expression was repressed in angiotensin converting enzyme inhibitor-treated samples. These genes, activated the hydroxylation of proline or lysin residues of procollagen mediated by 2-oxoglutaric acid and O_2,_ produce succinic acid and CO_2_. Metabolic analysis demonstrated the concentration of succinic acid was significantly increased in CHF samples compared with normal LV samples. Fibrosis was induced in human cardiac fibroblasts by TGF-ß1 treatment. After treatment, the gene and protein expressions of 2OGD, the concentration of succinic acid, and the oxygen consumption rate were increased compared with no treatment. This is the first study to show that collagen-related 2OGD genes contribute to HF during the induction of fibrosis and might be potential therapeutic targets for fibrosis and HF.

## Introduction

Myocardial interstitial fibrosis (MIF), characterized by the diffuse and disproportionate accumulation of collagen in the myocardial interstitium, can be characterized into two forms: replacement fibrosis and reactive fibrosis^1^. During replacement fibrosis, small foci of dead cardiomyocytes are replaced by MIF in regions where cells have fallen off, including areas of myocardial infarction and myocarditis, leading to the formation of microscars. In contrast, during reactive fibrosis, hypertension and valvular disease lead to chronic load, irritation, and inflammation, which continuously activate fibroblasts, promoting interstitial reactive fibrosis in the absence of cell shedding. Various factors including inflammatory cytokines and chemokines, reactive oxygen species, mast cell-derived proteases, endothelin-1, the renin/angiotensin/aldosterone system, matricellular proteins, and growth factors including TGF-ß and PDGF, are involved in fibrosis^2^. The progression of MIF causes cardiac dysfunction associated with decreased myocardial compliance leading to heart failure, lethal arrhythmia, and sudden death; therefore, the suppression of MIF is clinically important.

Animal models are commonly used to assess the therapeutic and/or adverse effects of experimental treatments. Rapid right ventricular pacing was previously reported to induce a dog model of congestive heart failure (CHF). Of note, the pathogenesis of CHF in dogs has a greater similarity to that in humans compared with mouse CHF models. Previously, we investigated the cellular and molecular alterations present in cardiac tissues during CHF^3–6^. Other proteomic and transcriptomic analyses demonstrated global changes that occur in cardiac tissues during CHF^7,8^.

In this study, we analyzed fibrosis during CHF using transcriptomic data and found that collagen-related 2-oxoglutarate-dependent dioxygenase (2OGD) genes enhanced CHF. This is the first study to show that 2OGD genes involved in collagen stabilization are related to fibrosis.

## Results

### RNA-seq analysis of CHF

**Figure 1:**
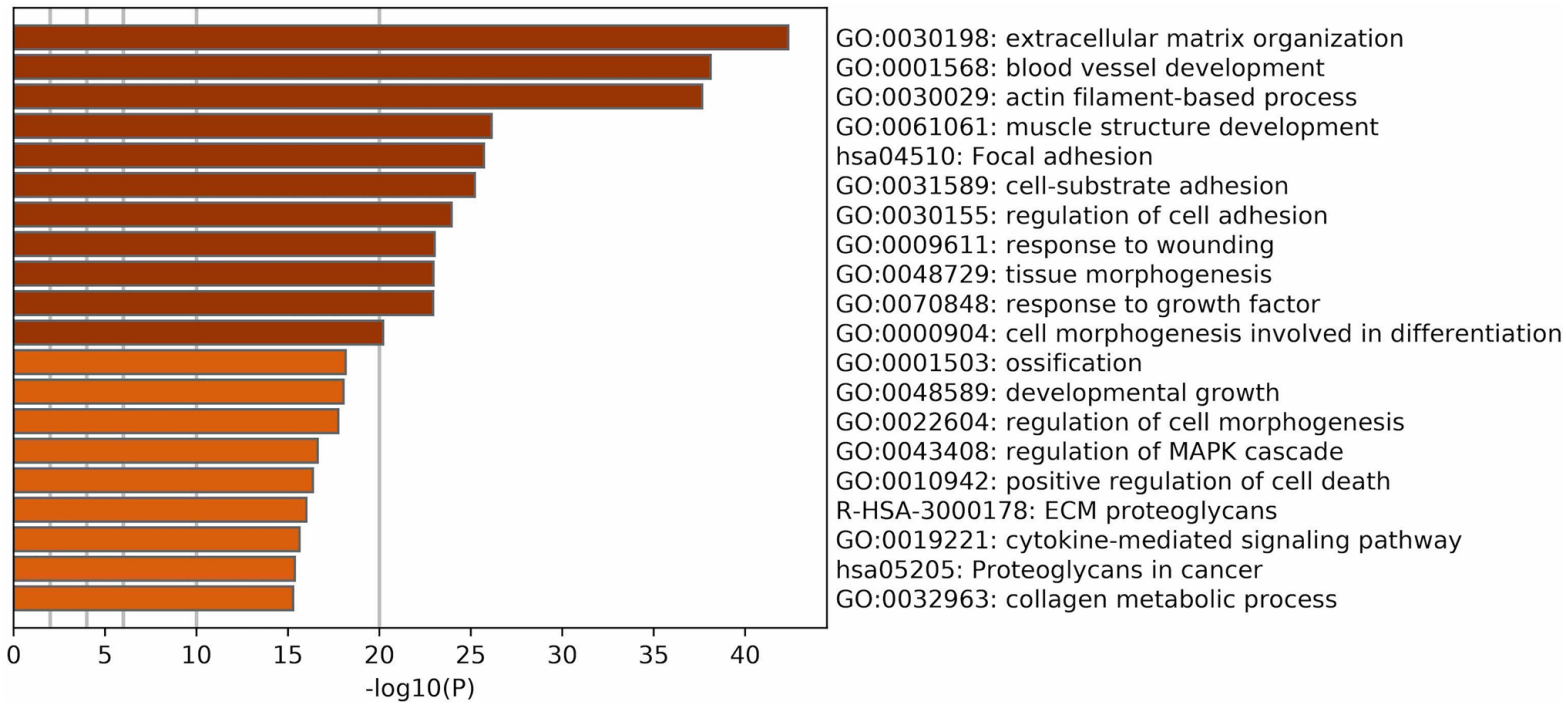
Enrichment_Heatmap of the top 20 significantly enriched pathways in CHF vs normal dogs. Enrichment analysis using Metascape was performed to input the 1729 DEGs and homologenes of *homo sapiens* (1557 genes) were used. GO: 0030198: extracellular matrix organization was the top pathway.

We performed digital transcriptome analysis to study the gene expression in the left ventricles (LV) of three normal control and six CHF dogs. RNA-seq analysis was performed using ELAND_RNA in CASAVA software and the CanFum3.1 dog genome database as a reference. A comparative analysis between normal control and CHF dogs was performed. We identified 1729 differentially expressed genes (DEGs) (Supplementary Table S1). Enrichment analysis using Metascape^9^ was performed by inputting 1729 DEGs and HomoloGene of *homo sapiens* (1557 genes). The top 20 clusters by pathway and process enrichment analysis are shown in Fig. 1. Overall, 150 DEGs were listed in the top pathway, gene ontology (GO): 0030198: extracellular matrix organization (Supplementary Table S2). This pathway is closely related to fibrosis and contains many collagen-related genes. In addition, GO: 0032963: collagen metabolic process was one of the top 20 clusters. We picked up 52 collagen-related DEGs from GO: 0030574: collagen catabolic process, 0030199: collagen fibril organization, 0032964: collagen biosynthetic process, 0032963: collagen metabolic process, and 0032967: positive regulation of collagen biosynthetic process in addition to GO: 0032963 and GO: 0032963 (Table 1). The accumulation of collagen during fibrosis requires the upregulated gene expression of COL genes as well as genes associated with collagen maturation. The collagen-related DEGs contained many genes in the 2OGD superfamily including *P3H1*, *P3H2*, *P3H4*, *P4HA2*, *PLOD1* and *PLOD3*, which are involved in collagen stabilization^10^.

**Table 1:** List of collagen-related DEGs between the LVs of CHF and normal dogs.

Gene symbol	CHF*	Normal*	log2(fold_change)**	q_value
*ADAM15*	10.028	4.74422	− 1.07979	0.021608
*ADAMTS14*	1.03495	0.235043	− 2.13856	0.001665
*ADAMTS2*	6.88804	2.10705	− 1.70887	0.001665
*BMP4*	4.16149	1.8686	− 1.15515	0.008824
*COL14A1*	18.6498	10.6494	− 0.808386	0.03704
*COL15A1*	47.1968	17.0654	− 1.46761	0.001665
*COL16A1*	2.9161	0.638799	− 2.19061	0.001665
*COL1A1*	60.9981	8.96067	− 2.76709	0.001665
*COL1A2*	63.7734	21.7366	− 1.55282	0.001665
*COL3A1*	174.769	39.6591	− 2.13972	0.001665
*COL4A1*	160.415	49.5708	− 1.69424	0.001665
*COL4A2*	107.406	29.6729	− 1.85585	0.001665
*COL5A1*	28.4263	6.98624	− 2.02464	0.001665
*COL5A2*	30.3133	13.5657	− 1.15999	0.001665
*COL5A3*	15.6045	5.60787	− 1.47644	0.001665
*COL6A1*	60.8866	15.8359	− 1.94292	0.001665
*COL6A3*	34.3907	13.8888	− 1.3081	0.001665
*COL8A1*	17.5812	7.17859	− 1.29226	0.001665
*COL9A2*	1.97375	0.407975	− 2.27439	0.001665
*CREB3L1*	3.71073	1.12514	− 1.7216	0.001665
*CTSK*	93.7015	47.8286	− 0.9702	0.005204
*CYGB*	11.1117	4.69163	− 1.24391	0.047176
*DPT*	150.923	81.6368	− 0.886519	0.001665
*ERRFI1*	16.5248	5.83304	− 1.50231	0.001665
*FAP*	10.4006	4.88204	− 1.0911	0.002981
*FMOD*	5.84089	2.2376	− 1.38423	0.001665
*FURIN*	23.7253	12.1107	− 0.970141	0.002981
*ID1*	14.6862	5.86282	− 1.3248	0.001665
*IL6*	14.1681	5.92985	− 1.25658	0.001665
*LOXL1*	11.2484	4.88911	− 1.20207	0.001665
*LOXL2*	24.8407	6.14567	− 2.01506	0.001665
*MFAP4*	20.6492	3.54362	− 2.54279	0.001665
*MMP14*	7.29472	3.37362	− 1.11255	0.001665
*MMP2*	22.0856	8.96237	− 1.30115	0.001665
*MMP23B*	4.07986	0.895127	− 2.18835	0.001665
*MMP28*	24.6738	9.85057	− 1.3247	0.001665
*MRC2*	8.24327	2.52699	− 1.7058	0.001665
*P3H1*	9.09977	5.16566	− 0.816878	0.035179
*P3H2*	3.21443	1.55853	− 1.04437	0.018145
*P3H4*	3.44936	1.12133	− 1.62112	0.001665
*P4HA2*	15.1185	8.1194	− 0.89687	0.015
*PDGFRB*	21.9649	12.6191	− 0.799589	0.024021
*PLOD1*	21.5431	7.74972	− 1.47501	0.001665
*PLOD3*	18.7454	9.0133	− 1.05641	0.001665
*RCN3*	6.93126	2.46855	− 1.48945	0.001665
*SERPINH1*	88.3682	36.9032	− 1.25978	0.001665
*TGFB1*	16.0169	8.47875	− 0.917672	0.01927
*TGFB2*	9.41672	3.85014	− 1.29031	0.002981
*TNS2*	24.3625	9.10933	− 1.41925	0.001665
*TNXB*	9.79429	3.48436	− 1.49105	0.043103
*UCN*	1.33901	0	–	0.004141
*VIM*	229.953	136.618	− 0.751189	0.005204

*FPKM.

**log2(Normal FPKM/CHF FPKM).

### Confirmation of 2OGD gene expression in CHF

**Figure 2:**
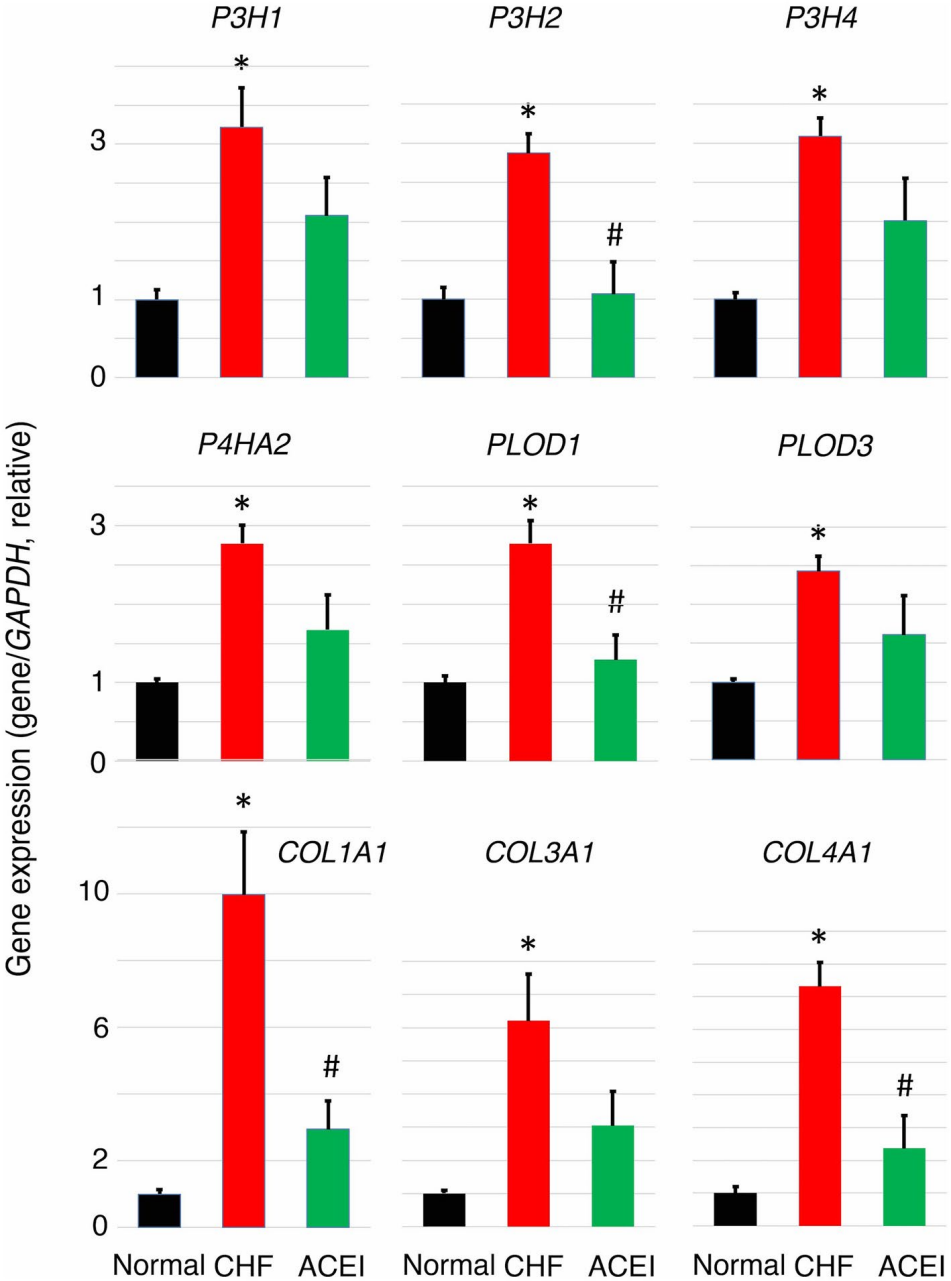
qRT-PCR analysis of 2OGD and collagen genes in LV samples from dog hearts. Quantitative RT-PCR was performed for each gene using four normal, six CHF, and four ACEI-treated LV RNAs from dog hearts. Gene expression was normalized to the *GAPDH* gene for normal samples. Error bars represent the standard errors. ANOVA was used for comparisons. p < 0.05, pairwise comparisons using *t*-tests with pooled SD vs normal (*) or CHF (#), respectively. One CHF sample was omitted because it had an abnormally high value. The expression of collagen-related 2OGD genes was increased in CHF samples compared with normal LV samples similar to collagen genes. The expressions of six 2OGD genes were repressed in ACEI-treated samples similar to collagen genes. These results suggested that collagen-related 2OGD genes contributed to CHF similar to collagen genes.

To investigate whether collagen-related 2OGD genes contributed to heart failure, we analyzed LV samples from our animal model by quantitative polymerase chain reaction (qPCR) (Fig. 2). We also analyzed the major collagen genes *COL1A1*, *COL3A1*, and *COL4A1*. The expressions of six 2OGD genes were significantly increased in CHF samples compared with normal LV samples similar to the collagen genes. Next, we analyzed the expressions of six 2OGD genes in angiotensin converting enzyme inhibitor (ACEI)-treated LV samples, which had improved CHF. The expressions of the six 2OGD genes were repressed in ACEI-treated LV samples similar to the collagen genes. These results suggested that collagen-related 2OGD genes contribute to CHF similar to the collagen genes.

### The enzyme activities of collagen-related 2OGD genes in CHF

**Figure 3:**
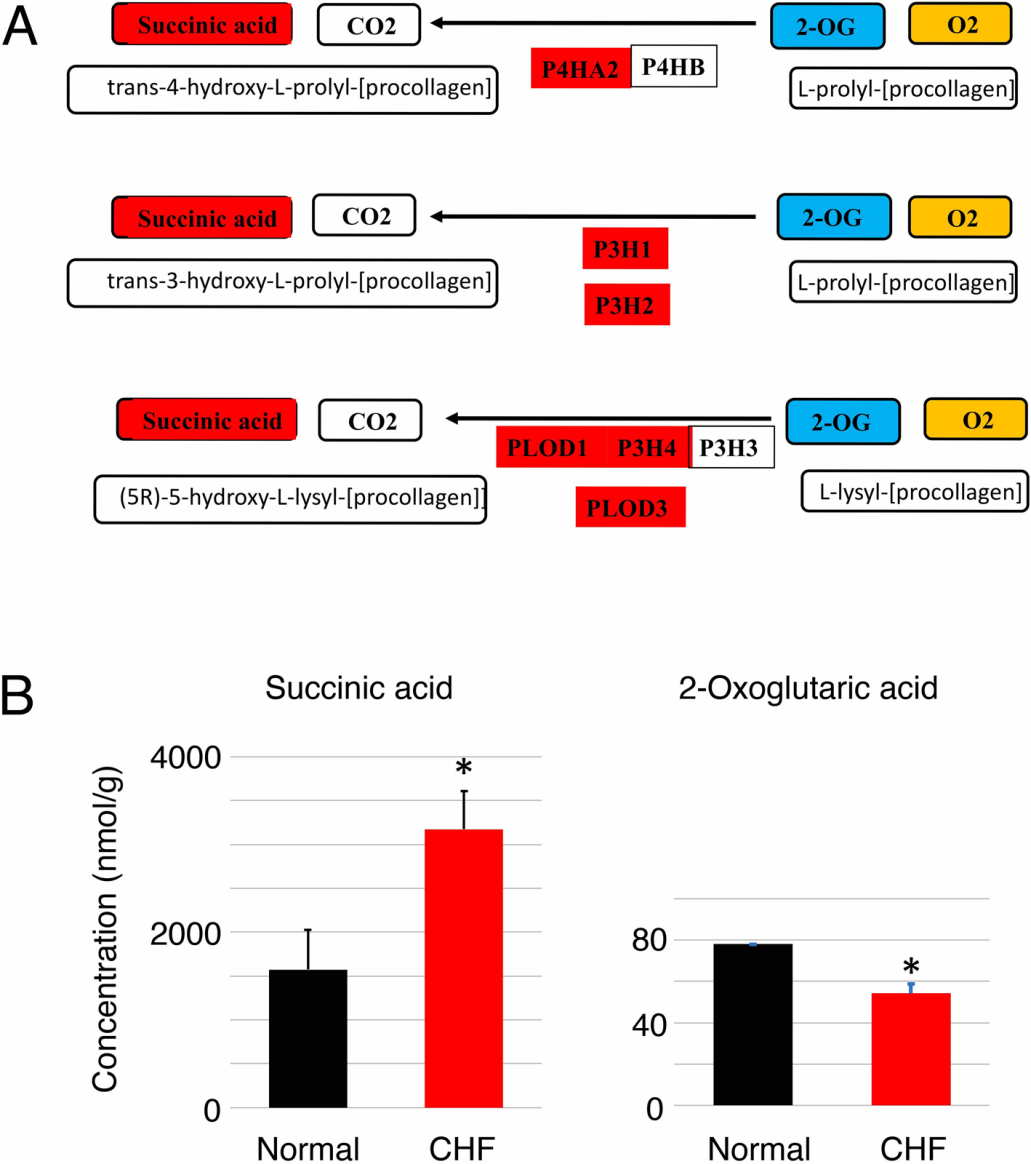
The enzyme activities of collagen-related 2OGD genes. (**A**) The enzyme reactions of collagen-related 2OGD genes. (**B**) Metabolic analysis of the enzyme activities of 2OGD genes in four normal and six CHF LV samples from dog hearts. Metabolic analysis showed that the concentration of succinic acid was significantly increased in CHF samples compared with normal LV samples, and that the concentration of 2-OG was decreased in CHF samples compared with normal LV samples, although two normal and one CHF LV samples were not determined due to low concentrations. These results suggested that the enzyme activities of collagen-related 2OGD genes were increased in CHF dogs compared with normal dogs.

The enzyme reactions of 2OGD genes are shown in Fig. 3A. These genes catalyze the reaction between the hydroxylation of proline or lysine residues of procollagen using 2-oxoglutaric acid (2-OG) and O_2_, which leads to the production of succinic acid and CO_2_. Metabolic analysis demonstrated the concentration of succinic acid was significantly increased in CHF samples compared with normal LV samples, and that the concentration of 2-OG was decreased in CHF samples compared with normal samples (Fig. 3B). These results suggested that the enzyme activities of the 2OGD genes were increased in CHF dogs compared with normal dogs.

### Confirmation of the expression of 2OGD in human cardiac fibroblasts (HCF)

**Figure 4:**
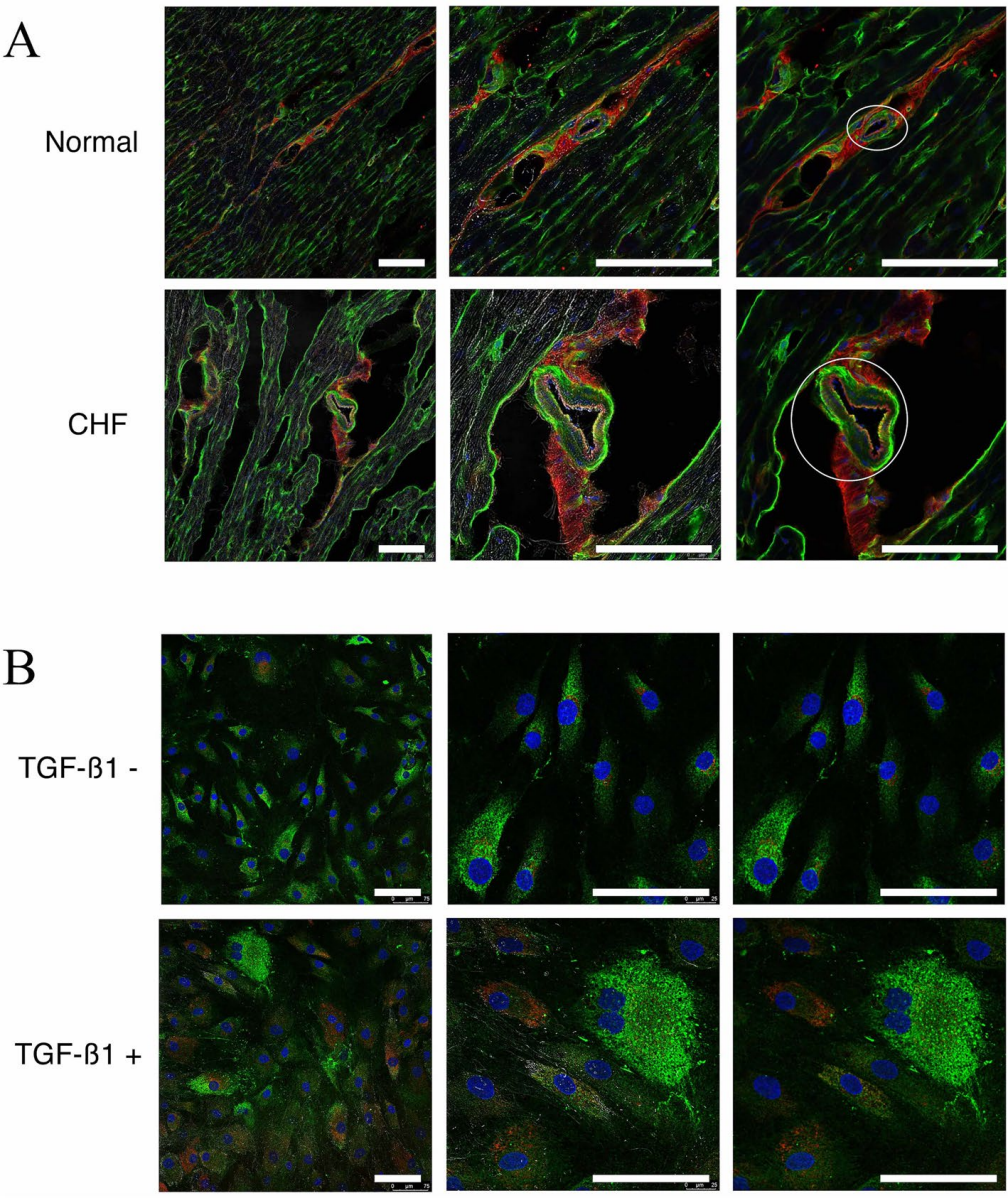
Immunofluorescence. (**A**) Photographs of LV tissues from normal and CHF dogs. Left photographs show staining by COL1A1 (red) and COL4A1 (green) antibodies, and DAPI (blue) with phase differences. Central photographs are the enlarged images of those on the left. Right photographs are the central images without phase differences. Blood vessels are enclosed in ovals. Blood vessels in LV tissues from CHF dogs were enlarged accompanied by vascular walls that had thickened by more than two-fold due to the accumulation of collagen. (**B**) Photographs of CHF cells with or without TGF-ß treatment. Left photographs show staining by COL1A1 (red) and COL4A1(green) antibodies, and DAPI (blue) with phase differences. Central photographs are the enlarged images of those on the left. Right photographs are the central images without phase differences. HCF with TGF-ß treatment were stained with anti-collagen antibodies similar to CHF LV samples. This result showed that TGF-ß1 treatment induced fibrosis in HCF. The white scale bars correspond to 100 μm.

**Figure 5:**
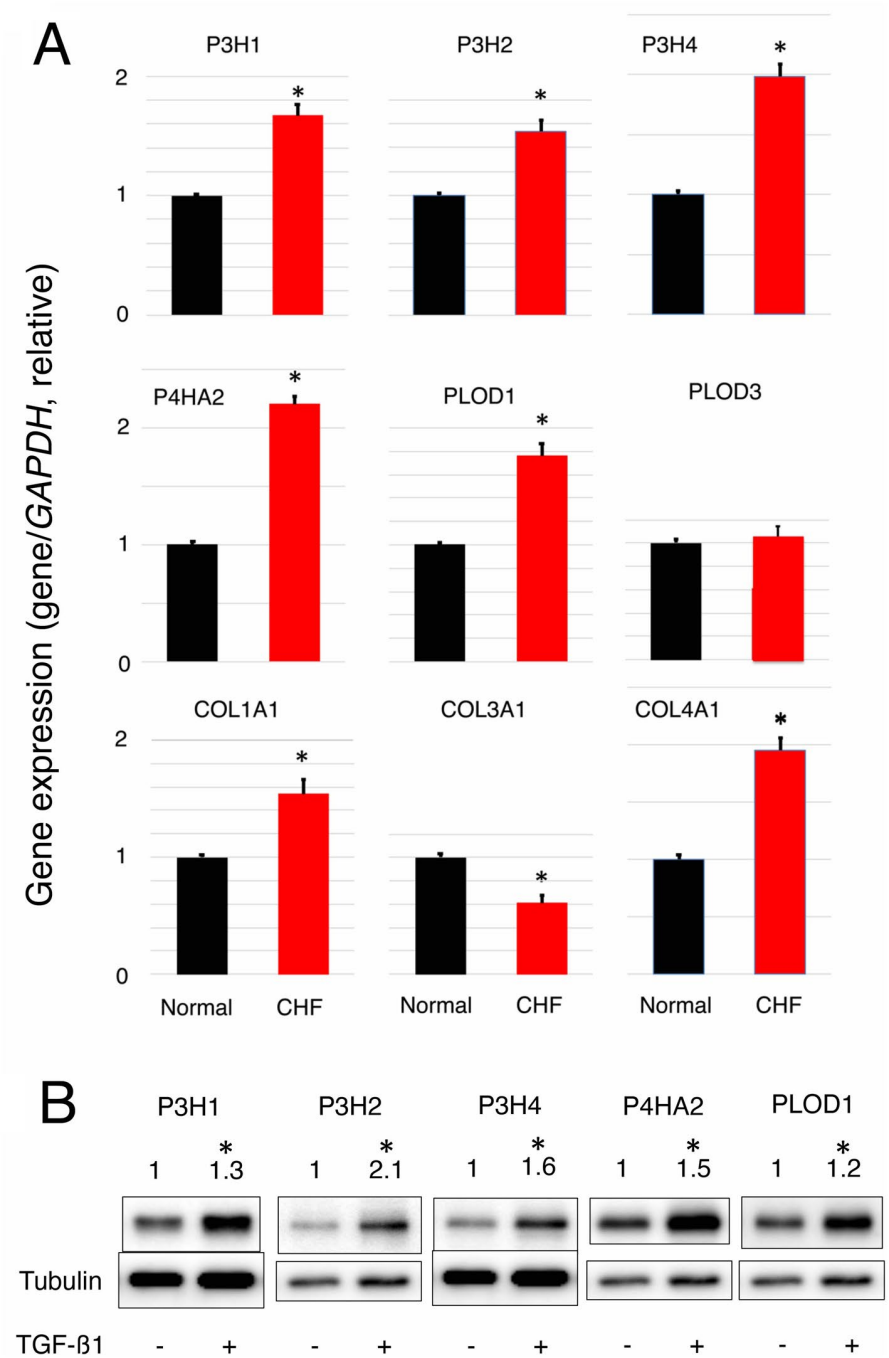
Expression of collagen-related 2OGD genes in HCF. (**A**) Quantitative RT-PCR was performed for each gene using HCF with or without TGF-ß treatment. Gene expression was normalized to the *GAPDH* gene in the normal samples. Error bars represent the standard errors. The values were derived from triplicate experiments. The Student’s *t*-test (two-tail) was used to compare differences between groups. Asterisks (*) indicate statistically significant differences (p < 0.05). (**B**) Immunoblot analysis was performed for each gene using HCF with or without TGF-ß treatment. Protein expression was normalized to α-tubulin in the normal samples. The values were derived from four replicate experiments. The Student’s *t*-test (two-tail) was used to compare differences between groups. Asterisks (*) indicate statistically significant differences (p < 0.05). The gene and protein expressions of five 2OGD except PLOD3 were significantly increased in HCF treated with TGF-ß compared with untreated cells.

We used rapid right ventricular pacing to generate a dog model of CHF^3^, which develops active fibrosis. Immunofluorescence analysis showed anti-human COL1A1 and COL4A1 antibodies cross-reacted with dog samples, and blood vessels in LV tissues from CHF dogs were enlarged, accompanied by vascular walls that had thickened by more than two-fold due to the accumulation of collagen (Fig. 4A). These results suggested the increase in collagen was related to the fibrosis of cardiac fibroblasts in the vascular walls. Therefore, to investigate whether collagen-related 2OGD genes contributed to CHF, we measured the expression of 2OGD in HCF. TGF-ß1 was used to induce fibrosis in HCF^11^, because the expression of *TGFB1* was significantly increased in CHF samples compared with normal LV samples (Table 1). HCF were treated with TGF-ß and stained with anti-collagen antibodies similar to CHF LV samples (Fig. 4B). We found that TGF-ß1 treatment induced fibrosis in HCF. The qPCR analysis of collagen-related 2OGD genes using HCF treated with TGF-ß (Fig. 5A) showed the expressions of five 2OGD genes were significantly increased in HCF compared with untreated HCF, except for *PLOD3,* which showed no difference in expression. In contrast, the expressions of *COL1A1*and *COL4A1* were significantly increased in HCF treated with TGF-ß. The expression of *COL3A1* was significantly decreased; however, the reason for this is unknown. The immunoblot analysis of collagen-related 2OGD using HCF (Fig. 5B and Supplementary Fig. S1) demonstrated the protein expressions of five 2OGD genes were significantly increased in HCF treated with TGF-ß compared with untreated HCF. However, PLOD3 expression was not detected in these experiments, because the antibodies for PLOD3 did not work. These results suggested that the increased expression of 2OGD contributed to fibrosis in cardiac fibroblasts in vascular walls.

**Figure 6:**
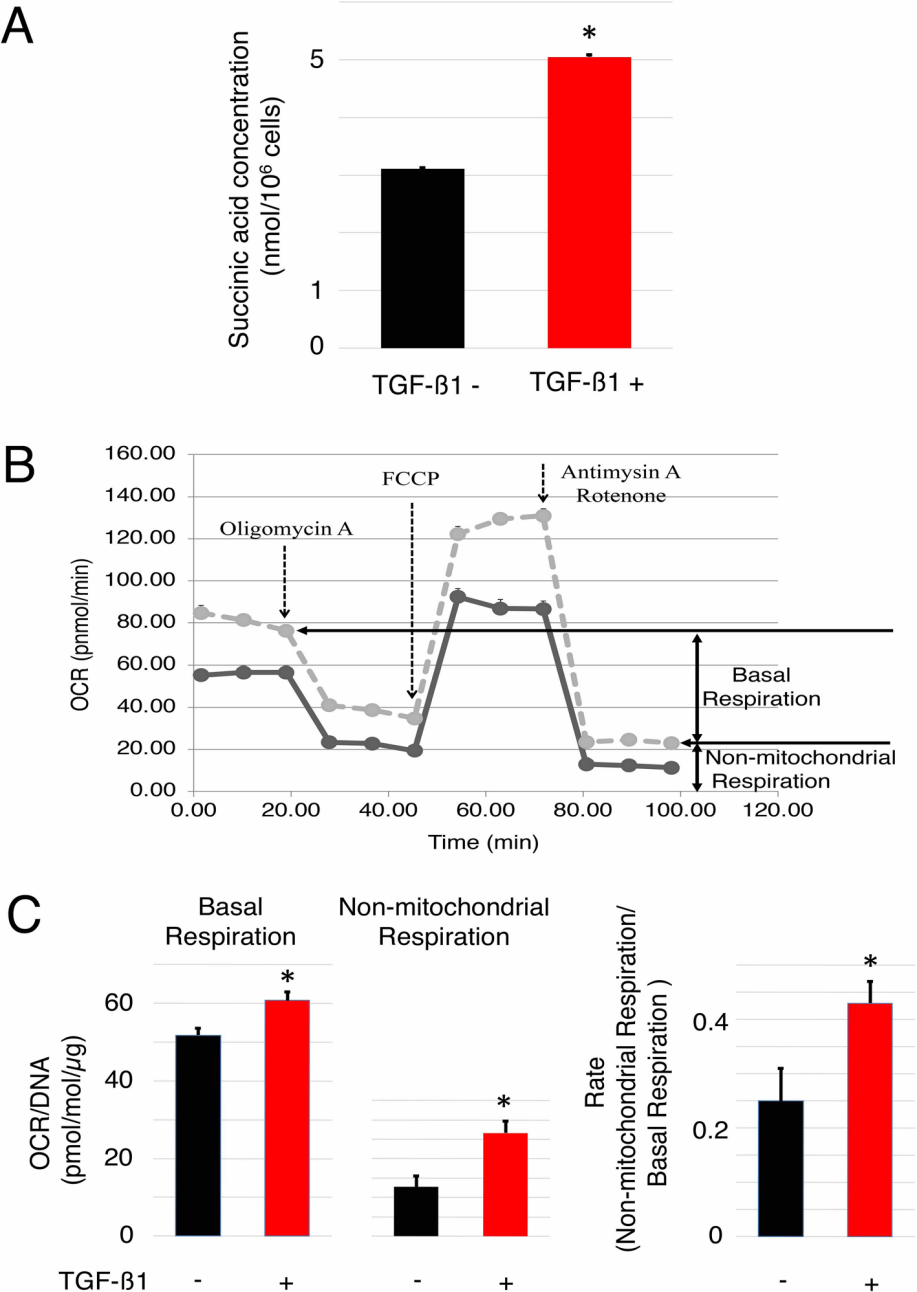
The enzyme activities of collagen-related 2OGD genes in HCF cells. (**A**) A quantitative assay of succinic acid in HCF was colorimetrically performed using a succinate assay kit. Error bars represent standard errors. The values were derived from triplicate experiments. The Student’s *t*-test (two-tail) was used to compare differences between groups. Asterisks (*) indicate statistically significant differences (p < 0.05). The quantitative assay of succinic acid showed that the concentration of succinic acid in HCF treated with TGF-ß was significantly increased compared with untreated cells. (**B**) Kinetic OCR responses of HCF treated with TGF-ß (dotted line) or untreated cells (solid line). Arrows indicate the timing of compound administration. OCR of non-mitochondrial respiration: OCR under rotenone, a mitochondrial complex I inhibitor and antimycinA, a mitochondrial complex III inhibitor, basal respiration: initial OCR minus OCR of non-mitochondrial respiration. (**C**) Basal respiration, non-mitochondrial respiration, and their ratio was calculated from the kinetic OCR responses of HCF (**B**). Basal respiration and non-mitochondrial respiration were normalized against DNA content, which reflected the cell number. Error bars represent the standard errors. The values were derived from five experiments. Asterisks (*) indicate statistically significant differences (p < 0.05). This result showed that the OCR was increased by non-mitochondrial respiration in HCF treated with TGF-ß. These results suggested that the enzyme activities of 2OGD genes, which consumed oxygen and produced succinic acid, were increased in HCF, which induced fibrosis after TGF-ß treatment.

To investigate whether the enzyme activities of 2OGD genes were increased in HCF treated with TGF-ß, we performed a quantitative assay to measure succinic acid and the oxygen consumption rate (OCR). The concentration of succinic acid in HCF treated with TGF-ß was significantly increased compared with untreated HCF (Fig. 6A). The OCR of non-mitochondrial respiration, induced by rotenone, a mitochondrial complex I inhibitor, and antimycin A, a mitochondrial complex III inhibitor, was significantly increased in HCF treated with TGF-ß compared with untreated HCF (Fig. 6B,C). Although basal respiration, the initial OCR minus the OCR of non-mitochondrial respiration, was also increased in HCF treated with TGF-ß compared with untreated HCF, the rate of non-mitochondrial respiration vs basal respiration was significantly increased in HCF treated with TGF-ß compared with untreated HCF. This suggested that the OCR was increased by non-mitochondrial respiration in HCF treated with TGF-ß and that the enzyme activities of 2OGD genes, which consumed oxygen and produced succinic acid (Fig. 3A), were increased in HCF, which promoted fibrosis induced by TGF-ß treatment.

## Discussion

Here, we demonstrated that collagen-related 2OGD genes contributed to CHF by inducing fibrosis in both in vivo and in vitro studies using a dog model and HCF, respectively. We found that collagen-related 2OGD genes stabilized collagen and oxygen consumption during the pathogenesis of CHF. During heart failure, myocardial ischemia or hypoxia occurs^12^ and cardiac fibroblasts may produce collagen by the induction of 2OGD expression in order to adapt to hypoxia. Myocardium in hearts is prone to more severe ischemia or hypoxia because of the active consumption of oxygen by cardiac fibroblasts during fibrosis. Furthermore, *LOXL1* and *LOXL2*, which were demonstrated to be collagen-related genes (Table 1), also consume oxygen during the process of cross-linking between collagen and elastin^13^. These genes may also cause severe ischemia in the myocardium of hearts. The active consumption of oxygen by cardiac fibroblasts during collagen biosynthesis may be a side effect of fibrosis in CHF.

We found that *PLOD3* and *COL3A1*expressions were increased in the LV of CHF dogs but not in HCF treated with TGF-ß. In addition to TGF-ß, many other factors are involved in fibrosis^2^. Therefore, *PLOD3* and *COL3A1* may be induced by other factors. Furthermore, many types of cells in addition to fibroblasts are present in the myocardium of the heart and might contribute to fibrosis and CHF. These cells might be involved in the control of collagen and collagen-related 2OGD gene expression. However, our study using HCF and TGF-ß generally reflected the action of collagen-related 2OGD genes on fibrosis and CHF.

We demonstrated that collagen-related 2OGD genes catalyzed the reaction of hydroxylation of proline or lysine residues of procollagen using 2-OG and O_2_, which led to the production of succinic acid and CO_2_ (Fig. 3A). We could not directly show an increase in the enzymatic activity of 2OGD in CHF. Therefore, we examined the concentration of succinic acid and 2-OG and the consumption of O_2_ in CHF dogs and HCF treated with TGF-ß (Figs. 3B, 6). As expected, the production of succinic acid and consumption of 2-OG were observed in CHF dogs. Furthermore, the production of succinic acid and consumption of O_2_ were observed in HCF treated with TGF-ß. These results demonstrated an increase in the enzymatic activity of collagen-related 2OGD in CHF.

This increase in the enzymatic activity of collagen-related 2OGD in CHF suggests that agents that inhibit the enzymatic reactions of collagen-related 2OGD may be potential therapeutic drugs to treat fibrosis and CHF. The prolyl hydroxylase domain-containing protein (PHD) of the 2OGD gene superfamily negatively regulates hypoxia-inducible factor (HIF). Recently, PHD inhibitors of HIF were developed as therapeutic drugs for renal anemia^14^. Furthermore, 2-OG analogs of these drugs cross-reacted with collagen-related 2OGD^15^. Therefore, 2-OG analogs may be potential therapeutic drugs for fibrosis and CHF. However, collagen is expressed ubiquitously in the body. Therefore, drugs specific for collagen-related 2OGD must be delivered directly to cardiac fibroblasts and myofibroblasts, which are activated by fibrosis. Periostin (*POSTN*) and α-smooth muscle actin (α-SMA, *ACTA2*) are marker proteins of activated cardiac fibroblasts and myofibroblasts, respectively^16,17^. Of note, *POSTN* and *ACTA2* were identified in 1729 DEGs between normal control and CHF dogs (Supplementary Table S1). Drugs specific for collagen-related 2OGD may act on fibrosis and CHF when delivered to activated cardiac fibroblasts through periostin.

In conclusion, this study demonstrated that collagen-related 2OGD genes contributed to CHF via the induction of fibrosis by the active consumption of oxygen and might be potential therapeutic targets for fibrosis and CHF.

## Methods

### Animal and sample preparation

All animal experiments were conducted according to the Guide for the Care and Use of Laboratory Animals (Department of Health and Human Services, National Institutes of Health, Publication no. 86-23), and Animal Research Reporting of In Vivo Experiments (ARRIVE) guidelines at the time when the animal experiments were performed, and approved by the Committee of Research Center for Animal Life Science at Shiga University of Medical Science. CHF was induced by rapid right ventricular pacing (240 beats per minute, 28 days) in beagles (Kitayama Labes Co., Ltd., Nagano, Japan), as described previously^3,5^. Dogs were randomly divided into three groups as follows: (1) the ACEI group (n = 6) received enalapril (1 mg/kg per day orally once a day); (2) the CHF group (n = 6) received only placebo and constituted time controls; and (3) the normal group (n = 6) received the same operation without pacing. ACEI treatment commenced on day 8 after the initiation of pacing and continued for 3 weeks. On the 28th day after the initiation of pacing, blood was collected for the hormonal assays of plasma atrial natriuretic peptide, aldosterone concentrations, and renin activity as previously described^3^. Then, a high-fidelity micromanometer catheter (SPC-350, Millar Instruments Inc, Huston, TX, USA) was placed into the LV under anesthesia and the mean arterial pressure, LV end-diastolic pressure (LVEDP), and cardiac output were measured. Echocardiography was performed and the LV end-diastolic diameter and % fractional shortening were measured (Supplementary Table S3). After the in vivo measurements were completed, the dogs were deeply anesthetized by intravenous injection with pentobarbital sodium (25 mg/kg) and euthanized by bleeding. The heart was rapidly removed and transmural sections of the left ventricle anterior free wall were frozen in liquid nitrogen and stocked at − 80 °C.

### Cell culture

HCF were purchased from PromoCell GmbH (Heidelberg, Germany) and maintained in Fibroblast Growth Medium with Supplement Mix (PromoCell GmbH) at 37 °C in a humidified 5% CO_2_ atmosphere. Treatment with 10 ng/mL human transforming growth factor-beta 1 (TGF-ß1; #100-21, PeproTech, New Jersey, USA) was performed for 5 days after 24 h pre-culture seeding at 3 × 10^4^ cells per 1 mL concentration in tissue culture flasks (Techno Plastic Products, Trasadingen, Switzerland) and cell culture plates (Thermo Fisher Scientific, Waltham, MA, USA).

### RNA preparation

Total RNA was extracted from the frozen heart LV muscles of normal control, CHF, and drug treated dogs using a Trizol Plus RNA Purification kit (Thermo Fisher Scientific). Total RNA from HCF was extracted using an RNA Purification kit (Thermo Fisher Scientific). For RNA-Seq, total RNA was quantified by an Agilent 2100 bioanalyzer (Agilent, Santa Clara, CA, USA) following the manufacturer’s instructions. The RNA Integrity Numbers of all prepared total RNA samples were ≥ 8.

### Genome analyzer sequencing

Template molecules for high throughput DNA sequencing were prepared from total RNA using an mRNA-Seq Sample Preparation Kit (Illumina, San Diego, CA, USA) following the manufacturer’s protocol. The library was quantified using an Agilent 2100 bioanalyzer. Each library (8 pM) was subjected to cluster amplification on a Single Read Flow Cell v4 using a cluster generation instrument (Illumina). Sequencing was performed on a Genome Analyzer GAIIx with 37 cycles, using Cycle Sequencing v4 reagents (Illumina). Image analysis and base calling were performed using Real Time Analysis version 1.13 (Illumina). Sequence libraries for each sample were processed using CASAVA Software 1.8.2 (Illumina) to produce 35-bp sequence data in fastq format. Fastq data were previously deposited in the DNA Data Bank of Japan (DDBJ) under accession number DRA005850. The datasets generated and/or analyzed during the current study are available in the DDBJ repository, accession number DRA005850.

### Data analysis

The fastq files of DRA005850 were processed by Cutadapt version 1.2.1^18^ with option –q 30. We removed reads shortened than 35-bp using Cutadapt. Trimmed reads for each sample were aligned to the reference genome (CanFam3.1) by TopHat version 2.0.10^19^ on the default setting, except for option –G. Differential gene expression analysis was performed by Cufflinks^20^ with option -g and focused on the contrast of normal control and CHF groups. Cuffmerge was used to merge the assembled transcripts into a consensus gene track from all mapped samples with options –s and –g. Then, Cuffqunt was used for quantification using option –M. Mitochondria genes, immunoglobulins, and HLA were removed. Cuffdiff was used to identify DEGs and transcripts between the normal and CHF groups. Genes and transcripts were identified as being significantly differentially expressed when they had q-values < 0.05 as calculated by the Benjamin-Hochberg FDR correction^20^. Values of fragments per kilobase of exon per million fragments mapped (FPKM) values were converted from count values to compare expression levels between genes. Enrichment analysis was performed using Matascape^7^.

### Quantitative reverse-transcription–polymerase chain reaction (qRT-PCR)

Quantitative RT-PCR was performed using the LightCycler 480 SYBG Master I Mix and LightCycler 480 System II (Roche Diagnostics, Mannheim, Germany). Gene expression was normalized to the *GAPDH* gene. Primer sequences are listed in Supplementary Table S4. All quantification analyses were performed in triplicate.

### Antibodies

The mouse monoclonal antibodies COL1A (COL-1) (diluted 1:500, sc-59772) and LLH1 (B-5) (diluted 1:500, sc-59772; PLOD1) were purchased from Santa Cruz Biotechnology (Santa Cruz, CA, USA). The rabbit polyclonal antibodies COL4A (diluted 1:1000, ab6586) and P3H1 (diluted 1:500, ab154799) were purchased from Abcam, (Cambridge, UK). The rabbit polyclonal antibodies P3H2 (diluted 1:500, 15723-1-AP), P3H4 (diluted 1:500, 15288-1-AP), and P4HA2 (diluted 1:500, 13759-1-AP) were purchased from Proteintech (Rosemount, IL, USA). The mouse monoclonal antibody anti-α-tubulin (DM1A) (diluted 1:1000, #T9026) was purchased from Sigma-Aldrich (St. Louis, MO, USA). The anti-mouse Ig (diluted 1:200, Alexa Fluor™ Plus 594) and anti-rabbit Ig (diluted 1:200, Alexa Fluor™ Plus 488) were purchased from Thermo Fisher Scientific.

### Immunofluorescence

Surgical specimens of LV were frozen and stored in a deep freezer. The frozen samples were embedded in O.C.T. compound (Sakura Finetek Japan co., Ltd., Tokyo, Japan) and stored at − 20 °C after quick freezing. The embedded samples were serially sliced into 10-µm sections using a cryostat (CM3050 S, LEIKA Biosystems, Wetzlar, Germany). After dewaxing, the sections were blocked for 1 h with phosphate buffer saline (PBS) containing 2% bovine serum albumin (BSA). Then, they were incubated overnight at 4 °C with primary antibodies. Next, the sections were rinsed with PBS and incubated with secondary fluorescence-labeled antibodies at room temperature for 1 h. Glass slides were used to mount samples with ProLong™ Gold antifade reagent with DAPI (Thermo Fisher Scientific). HCF were cultured in 35-mm glass bottomed dishes (Matsunami Glass, Osaka, Japan). For immunofluorescence staining, medium was removed and the cells were fixed with 4% paraformaldehyde for 5 min, permeabilized with 0.1% Triton X-100 for 5 min, and then blocked with 2% BSA for 30 min. The cells were washed three times with PBS before being incubated with primary antibody overnight. Cells were then incubated with a secondary fluorescence-labeled antibody for 1 h. The nucleus was stained with DAPI (NucBlue Fixed Cell Stain ReadyProbes regent, Thermo Fisher Scientific).

### Immunoblotting

Cells were lysed in Laemmli-SDS buffer, subjected to SDS–polyacrylamide gel electrophoresis, and electro-transferred to membrane filters (Immuno-Blot PVDF membranes, Bio-Rad Laboratories, Richmond, CA, USA). The filters were incubated overnight with primary antibody in TBS-T containing 2% BSA and incubated for 1 h with horseradish peroxidase-conjugated anti-mouse or anti-rabbit secondary antibody (Cell Signaling Technology, Danvers, MA, USA) diluted 1:5,000 in TBS-T containing 2% BSA. Immunoreactivity was detected using the Luminata Classico Western HRP substrate (Millipore Corporation, Burlington, MA, USA) with LAS4000 (Fujifilm, Tokyo, Japan) and quantified with MultiGauge software (Fujifilm), using an anti-α-tubulin antibody as the internal control.

### Metabolic analysis

The quantitative measurements of succinic acid and 2-OG in LV samples were obtained from metabolic analysis data at the Human Metabolome Technologies (HTM, Tsuruoka, Japan), using LV samples from four normal and six CHF dogs. The quantitative measurement of succinic acid in HCF was performed using a Succinate Assay kit (Colorimetric) (ab204718, Abcam) following the manufacturer’s instructions. Colorimetry of succinic acid was determined at 450 nm, using an InfinitM200 (TECAN, Mannendorf, Switzerland). Quantification analyses were performed in triplicate.

### Measurement of the OCR

OCR measurements were performed using an XF24^e^ Extracellular Flux analyzer (Seahorse Bioscience, North Billerica, MA, USA) as previously described^21^. Briefly, HCF were plated on XF24 cell culture plates (Seahorse Bioscience) after 4 days of culture in Fibroblast Growth Medium with Supplement Mix with or without 10 ng/mL TGF-ß1 and then cultured for one day at 37 °C in a humidified incubator with 5% CO_2_. Prior to the assay, the growth medium in the wells of the XF cell plate was replaced with Fibroblast Growth Medium with Supplement Mix with or without 10 ng/mL TGF-ß1. The sensor cartridge was calibrated and the cell plate was incubated at 37 °C in a non-CO_2_ incubator for 1 h. All experiments were performed at 37 °C. After completion of the assay, DNA was extracted from cells in each well using a DNeasy Blood & Tissue Kit (Qiagen, Hilden, Germany). Then, the extracted DNA was measured using a Smart Spec 3000 (Bio-Rad Laboratories, Hercules, CA, USA) to assess cell numbers. The OCR was assayed using an XF Cell Mito Stress Test Kit (Seahorse Bioscience) following the manufacturer’s instructions. The following compounds were injected: oligomycin (1 µM), an ATP synthetase inhibitor; FCCP, (0.5 µM), an uncoupler reagent; rotenone (1 µM), a mitochondrial complex I inhibitor; and antimycin A (1 µM), a mitochondrial complex III inhibitor as previously described^22^.

### Statistical analysis

The data were reported as the means ± standard error (SE). The values were derived from at least three experiments. The Student’s *t*-test (two-tail) was used to compare differences between groups. One-way factorial analysis of variance (ANOVA), accompanied by pair-wise comparisons using *t-*tests with pooled standard deviation (SD) was used to compare the means of multiple groups using the free software R. A p-value < 0.05 indicated statistical significance.

## Supplementary Information

Supplementary Information.

## Data Availability

The datasets generated and/or analyzed during the current study are available in the DNA Data Bank of Japan (DDBJ) repository, accession number DRA005850.
