# Supplementary material for: Transcriptome analysis of a dog model of congestive heart failure shows that collagen-related 2-oxoglutarate-dependent dioxygenases contribute to heart failure
Source: Sci Rep. 2022 Dec 29;12:22569. doi: 10.1038/s41598-022-26717-7 (PMC9800379; doi:10.1038/s41598-022-26717-7)
Supplement: Supplementary file 1 — Supplementary Information. [file 41598_2022_26717_MOESM1_ESM.pdf]

Transcriptome analysis of a dog model of congestive heart failure shows that collagen-related 2-oxoglutarate-dependent dioxygenases contribute to heart failure.

Takahiro Isono, Takehiro Matsumoto, Masafumi Suzaki, Shigehisa Kubota, Susumu Kageyama, Akihiro Kawauchi, and Atuyuki Wada

Supplementary figure S1. Blots used in Figure 5B.

A. Photographs of original blot data.

Membrane1 first hybridization

Immunoblot

P3H4

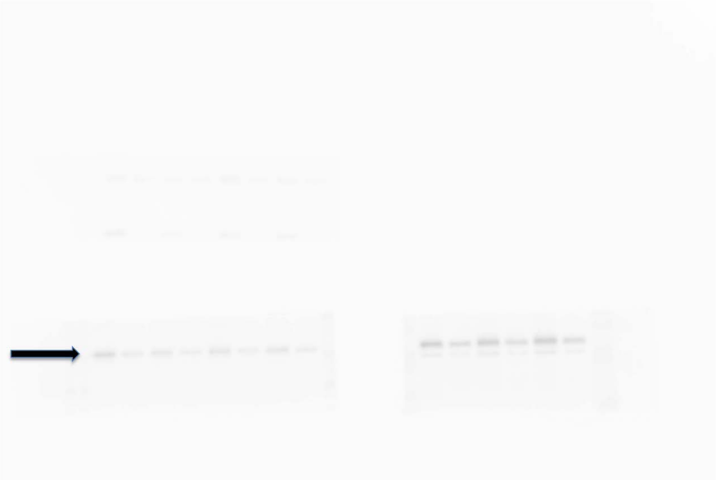

Membrane

P3H4

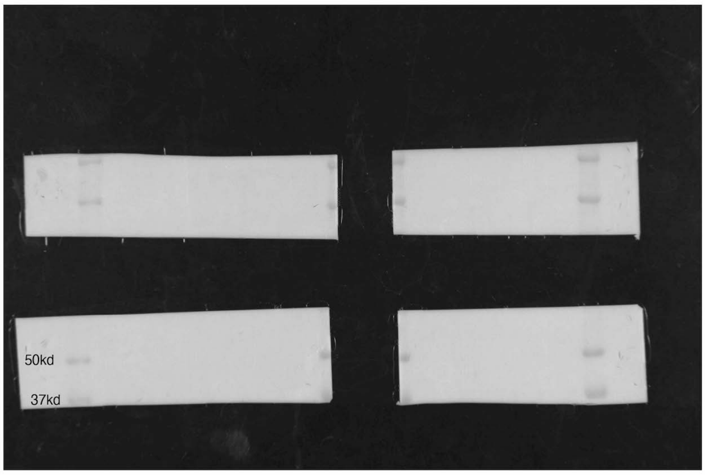

Overlay

P3H4

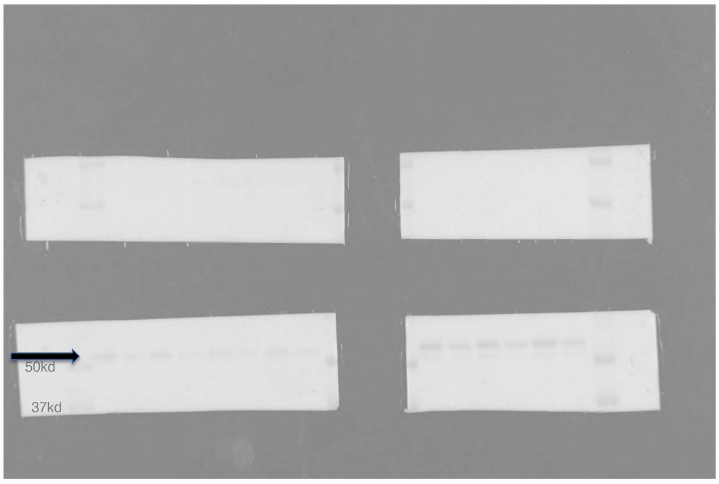

Membrane1 rehybridization

Immunoblot

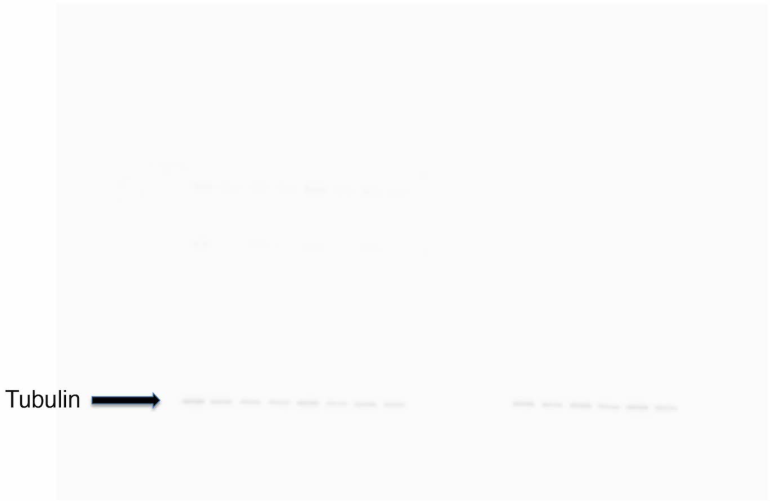

Membrane

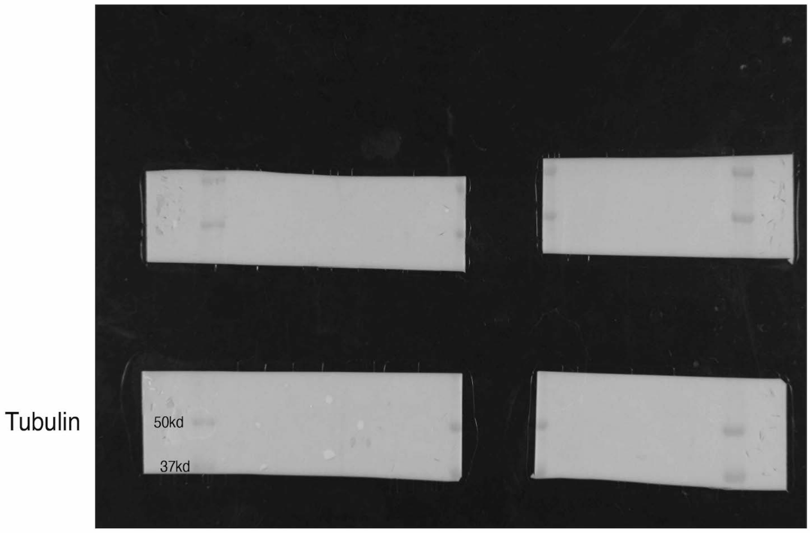

Overlay

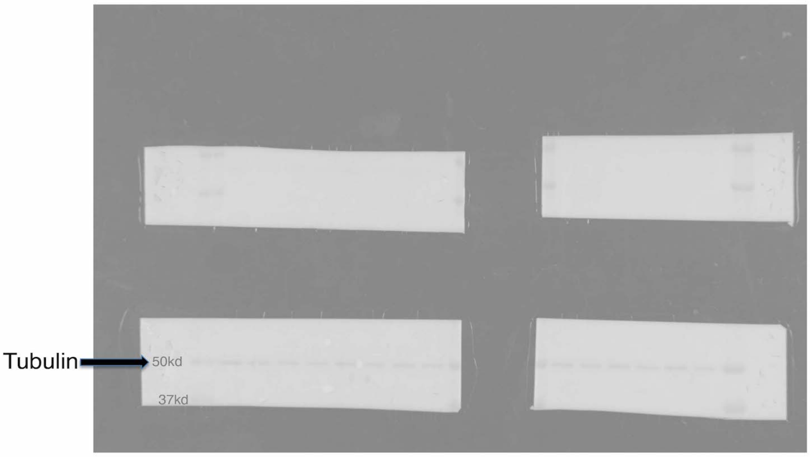

Membrane1 re-rehybridization

Immunoblot

P3H1

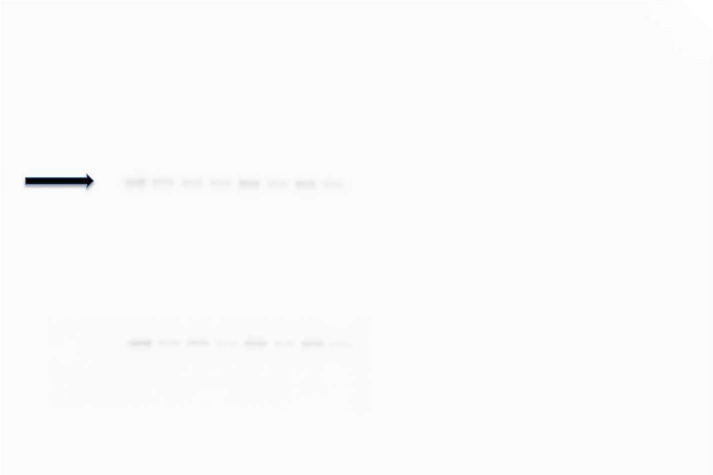

Membrane

P3H1

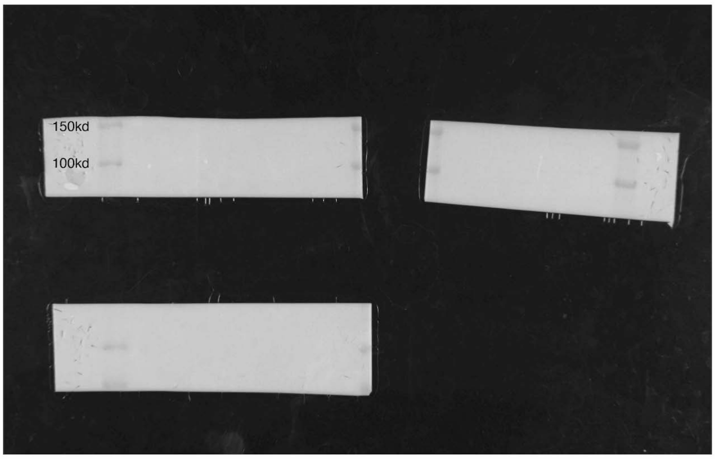

Overlay

P3H1

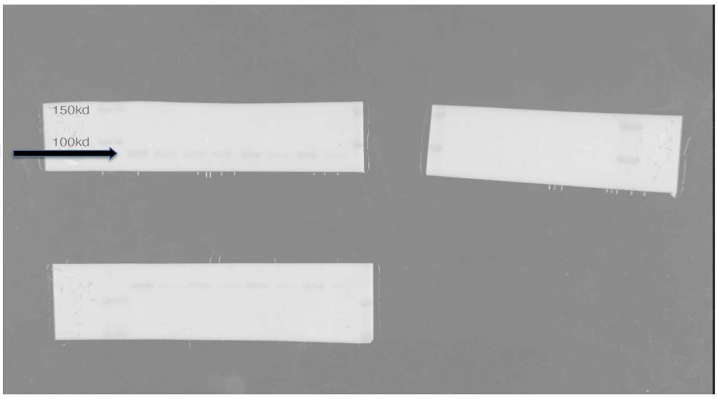

Membrane2 first hybridization

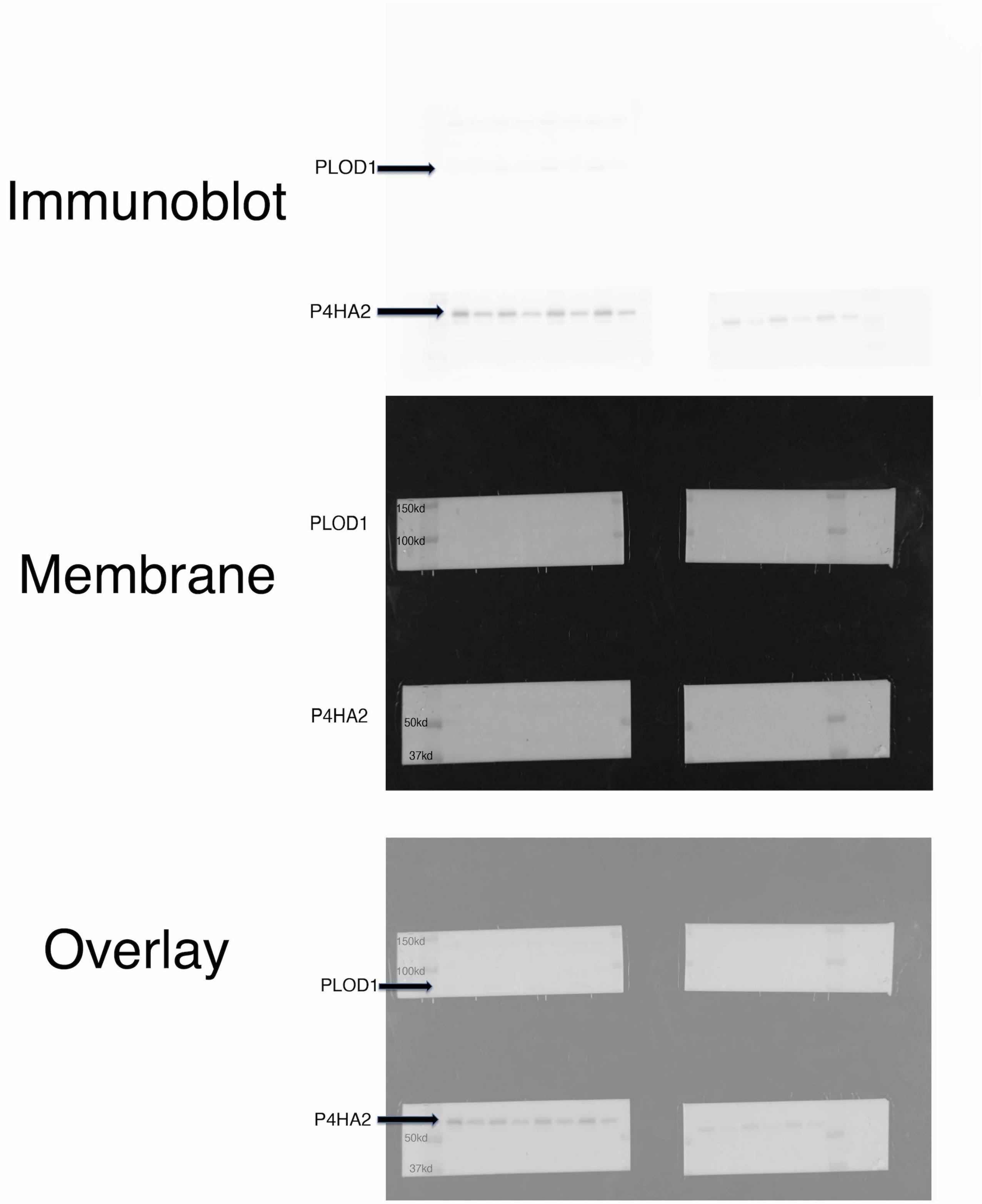

Membrane2 rehybridization

Immunoblot

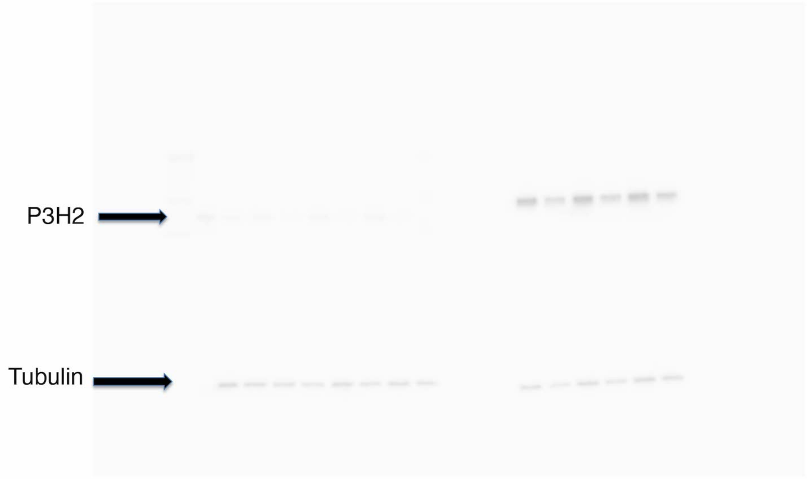

Membrane

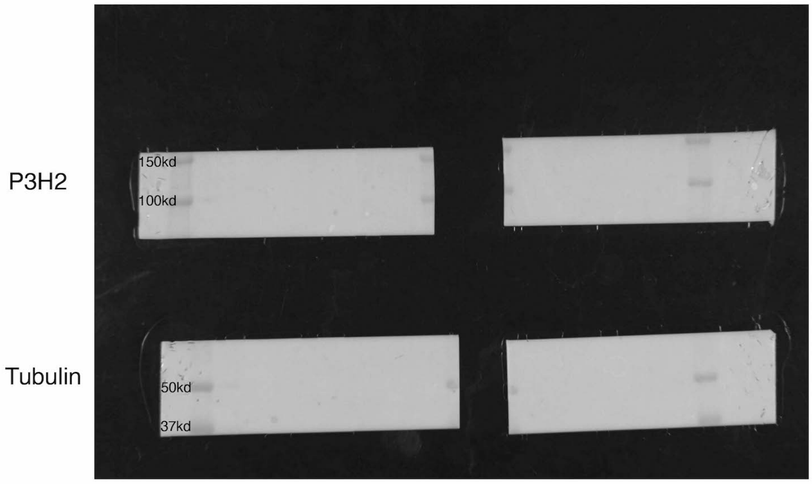

Overlay

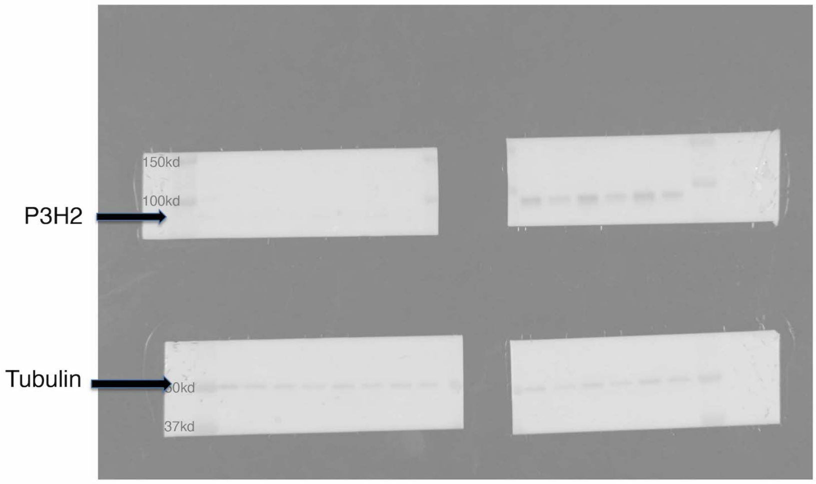

B. Photographs of trimmed original blot data.

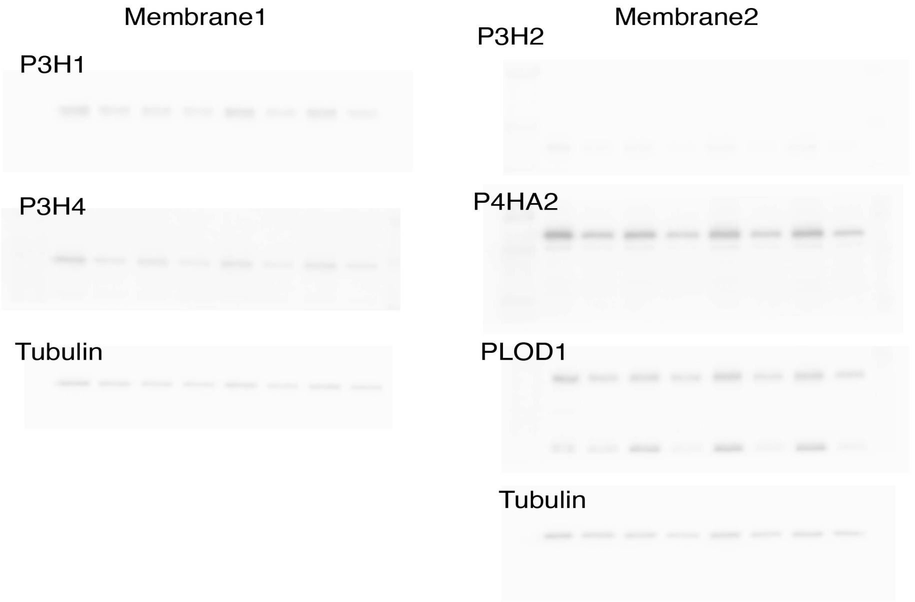

C. Photographs of blot data after contrast fitting.

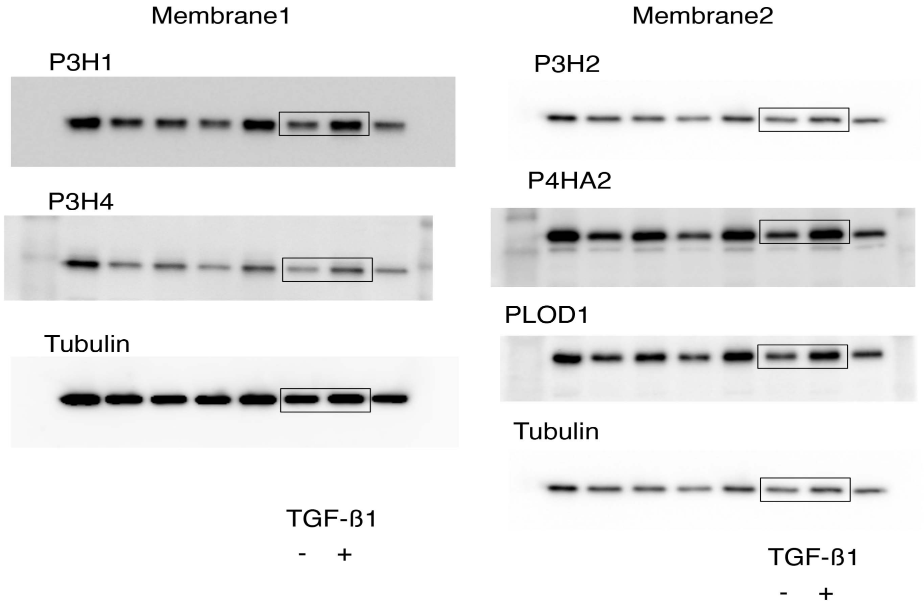

A. Photographs of original blot data. The protein blotting membranes were cut prior to hybridization with antibodies. Photographs of immunoblots, membranes, and the overlay of each hybridization are shown. LAS4000 data had a good signal-to-noise (SN) ratio in a short time. The original immunoblot data had a low signal and the membrane edges could not be shown. Therefore, photographs of the membranes, which were taken at the same time as immunoblot data, were added. These photographs show the membrane edges. The protein molecular size is shown on the membranes using a pre-stained protein marker (Bio-Rad Laboratories, Richmond, CA, USA). Then, photographs of the overlaid immunoblots and membranes were added to demonstrate the original blot data were correct.

B. Photographs of trimmed original blot data. Photographs of original blot data were cut and summarized. Blots of P3H1 and P3H4 were used for immunoblot analysis using Membrane1 paired with a tubulin blot. Blots of P3H2, P4HA2, and PLOD1 were used for immunoblot analysis using Membrane 2 paired with a tubulin blot.

C. Photographs of blot data after contrast fitting. Photographs used in Figure 5B were surrounded by squares and were treated to more contrast fitting.

Supplementary Table S1. List of DEGs between the LV of CHF and normal dogs.

Gene symbol	CHF*	Normal*	log2(fold_change)**	q_value
ENSCAFG00000048083	67.8077	1.59005	-5.4143	0.00166463
ENSCAFG00000037114	24.6412	0.984078	-4.64615	0.00166463
RIN1	2.75333	0.148017	-4.21734	0.00166463
AREG	1.16596	0.0862389	-3.75703	0.00799923
MEGF6	2.01653	0.173504	-3.53884	0.00166463
ENSCAFG00000029127	2.08057	0.186721	-3.47802	0.0204062
ADM5	3.2467	0.298918	-3.44115	0.0240207
ENSCAFG00000048643	33.5705	3.49119	-3.2654	0.00166463
ENSCAFG00000049023	3.19878	0.346997	-3.20453	0.00298108
KCNG1	0.826777	0.0958033	-3.10935	0.0156032
ENSCAFG00000043834,MIR23A,MIR24-2,cfa-mir-27a	41.7305	4.90406	-3.08905	0.00166463
CLDN5	19.124	2.24768	-3.08887	0.00166463
KRT80	7.9701	1.00919	-2.98141	0.00166463
ADAMTSL2	2.54029	0.324501	-2.9687	0.00166463
ENSCAFG00000043825	1.57738	0.204009	-2.95082	0.0245705
IER5L	1.84772	0.244078	-2.92033	0.0186537
FSTL3	33.2794	4.41547	-2.91399	0.00166463
ENSCAFG00000049840	8.17056	1.12817	-2.85646	0.00166463
ENSCAFG00000046086	1.19592	0.170053	-2.81407	0.0439079
CCDC85B	7.78532	1.12832	-2.78658	0.0052039
SLAMF8	1.65805	0.240389	-2.78605	0.011901
COL1A1	60.9981	8.96067	-2.76709	0.00166463
FTH1	159.566	23.5062	-2.76304	0.00166463
NGB	1.21958	0.180833	-2.75366	0.0288776
ENSCAFG00000047351	4.95546	0.738247	-2.74684	0.00166463
ENSCAFG00000049871	22.989	3.42967	-2.7448	0.00166463
FZD5	2.72177	0.406485	-2.74327	0.00298108
FIBIN	13.2134	1.98174	-2.73716	0.00166463
RAB20	2.63772	0.399611	-2.72262	0.030955
CSF3R	1.49089	0.227388	-2.71295	0.00166463
SCARF2	0.804128	0.124184	-2.69494	0.0411794
ENSCAFG00000028721	1.8269	0.287834	-2.66609	0.0348071
NEFH	3.59815	0.570869	-2.65602	0.00166463
PLEKHA4	0.739713	0.118986	-2.63618	0.00414075
RHBDL3	7.74096	1.26975	-2.60797	0.00166463
MFSD2A	1.22179	0.201241	-2.602	0.00298108
ENSCAFG00000018277	235.473	38.8154	-2.60086	0.00166463
TNFRSF12A	266.13	44.0181	-2.59596	0.00166463
ELN	44.997	7.47509	-2.58966	0.00166463
MYBL2	0.785896	0.130796	-2.58702	0.00709292
PIMREG	0.857437	0.142889	-2.58513	0.0328579
NCF4	1.02311	0.171831	-2.5739	0.0338795
ENSCAFG00000031437	1.82586	0.310551	-2.55567	0.0052039
MRPS34	12.9841	2.22705	-2.54354	0.00166463
MFAP4	20.6492	3.54362	-2.54279	0.00166463
ICAM4	8.97248	1.5448	-2.53809	0.00166463
GADD45G	14.0036	2.43934	-2.52124	0.00166463
BGN	59.8914	10.6424	-2.49253	0.00166463
SOX17	7.28726	1.30009	-2.48676	0.00166463
ENSCAFG00000022282,ENSCAFG00000026472	81.6809	14.7351	-2.47074	0.00166463

FAM110D	5.14925	0.936243	-2.45941	0.00166463
GPR84	1.88619	0.343312	-2.45788	0.0150001
ENSCAFG00000042712	0.959891	0.174746	-2.45761	0.0487642
C1QB	23.9998	4.39031	-2.45063	0.00166463
PRAG1	1.91739	0.350832	-2.45029	0.00166463
ISG20	5.37287	1.01472	-2.40461	0.00799923
SLCO2A1	7.50347	1.42993	-2.39162	0.00166463
CCN1	208.838	40.2285	-2.37609	0.00166463
C1QA	21.1481	4.11755	-2.36067	0.00166463
CHST1	3.41115	0.667138	-2.3542	0.00166463
DUSP5	6.86453	1.36736	-2.32776	0.00166463
CHI3L1	2.0237	0.403251	-2.32724	0.00618756
ENSCAFG00000014751	19.1303	3.81331	-2.32674	0.00166463
CD68	2.35399	0.469507	-2.32589	0.00618756
TUBB2B	2.77866	0.560328	-2.31004	0.00166463
DKK2	44.8756	9.10232	-2.30162	0.00166463
RHBDF2	4.2306	0.859295	-2.29964	0.00166463
CYP4F22	2.53639	0.516523	-2.29587	0.00166463
PHF21B	1.87096	0.385448	-2.27917	0.00166463
COL9A2	1.97375	0.407975	-2.27439	0.00166463
SOX4	1.94638	0.406289	-2.26021	0.0228621
TNF	1.89258	0.396493	-2.25499	0.0338795
LRWD1	30.7117	6.52056	-2.23572	0.0204062
ENSCAFG00000023691	29.5553	6.30502	-2.22885	0.0319719
OLFM2	2.14158	0.45779	-2.22591	0.0052039
SERPINA1	2.84797	0.61157	-2.21935	0.00166463
PGK2	1.53081	0.331404	-2.20764	0.0104658
LRRC25	3.71641	0.806552	-2.20407	0.00298108
CCDC85C	11.6327	2.54161	-2.19437	0.0181454
PHF19	0.765224	0.16741	-2.19249	0.0421371
COL16A1	2.9161	0.638799	-2.19061	0.00166463
ENSCAFG00000000641	226.88	49.7108	-2.1903	0.00166463
MMP23	4.07986	0.895127	-2.18835	0.00166463
SLC16A3	1.95996	0.43196	-2.18185	0.0111445
PI16	47.1303	10.3889	-2.18161	0.00166463
ENSCAFG00000038629	2.82243	0.62338	-2.17875	0.00799923
SFRP4	2.7967	0.622314	-2.16801	0.00166463
RUNX1	1.38581	0.311886	-2.15164	0.0192697
ANKRD33B	0.83439	0.188292	-2.14775	0.0104658
SLC11A1	2.08841	0.472472	-2.1441	0.00166463
ENSCAFG00000044253	2.15658	0.488458	-2.14244	0.0283656
COL3A1	174.769	39.6591	-2.13972	0.00166463
RAC2	3.13103	0.711071	-2.13857	0.0170082
ADAMTS14	1.03495	0.235043	-2.13856	0.00166463
IL15RA	0.810405	0.184869	-2.13214	0.026814
ENSCAFG00000044317	13.3755	3.0691	-2.1237	0.00166463
LTBP4	47.2743	10.8892	-2.11815	0.00166463
FOS	44.4495	10.2615	-2.11492	0.00166463
SOD3	5.68164	1.3145	-2.11179	0.00166463
ZYX	54.0502	12.5096	-2.11126	0.00166463
KLHL25	12.6991	2.93963	-2.11102	0.00166463
FOSL1	9.34628	2.16754	-2.10833	0.00166463
FAM43A	5.02791	1.169	-2.10469	0.011901

ENSCAFG00000031378	2.20882	0.513867	-2.10381	0.0175929
MKI67	0.964829	0.224862	-2.10124	0.00166463
HSPG2	42.8808	9.99567	-2.10096	0.00166463
CCN2	299.869	69.9535	-2.09986	0.00166463
C32H4orf54	2.15989	0.506186	-2.09322	0.00166463
HCK	1.36659	0.32075	-2.09106	0.008824
NFASC	1.5967	0.374777	-2.09099	0.00166463
ITGB2	1.40682	0.334108	-2.07405	0.00166463
KIF26B	0.769904	0.183785	-2.06666	0.00166463
LIMD2	2.96541	0.709198	-2.06397	0.00298108
ROBO3	1.89922	0.455628	-2.05948	0.0052039
TBC1D2	0.884374	0.21292	-2.05435	0.0198195
IER3	157.149	37.8984	-2.05192	0.00166463
HELZ2	3.27836	0.790958	-2.0513	0.00166463
HDAC4	5.75575	1.41054	-2.02876	0.00166463
ENSCAFG00000037587	1.72278	0.422482	-2.02778	0.0313973
ENSCAFG00000010825	1.12253	0.275608	-2.02607	0.00166463
COL5A1	28.4263	6.98624	-2.02464	0.00166463
NECTIN1	3.69226	0.909829	-2.02084	0.00166463
KCTD12	2.84856	0.702371	-2.01993	0.0111445
ENSCAFG00000016333	21.2557	5.24625	-2.01849	0.00166463
PKP3	0.650726	0.160934	-2.01558	0.0135044
LOXL2	24.8407	6.14567	-2.01506	0.00166463
FLNC	757.254	187.868	-2.01106	0.00166463
HIVEP3	1.2334	0.307338	-2.00475	0.00166463
DOHH	2.74263	0.688889	-1.99321	0.00709292
ENSCAFG00000029470	23.494	5.93246	-1.98559	0.00166463
XIRP1	559.201	141.273	-1.98488	0.00166463
MMRN2	8.25965	2.09059	-1.98217	0.00166463
KLHL29	1.75842	0.446345	-1.97805	0.00166463
LHX6	3.46682	0.88422	-1.97114	0.00166463
FKBP10	4.94872	1.26332	-1.96983	0.00166463
DEPP1	18.4901	4.73921	-1.96404	0.0408367
AEBP1	13.8489	3.5552	-1.96177	0.00166463
PFKP	36.1426	9.29098	-1.9598	0.00166463
MYC	24.6492	6.36704	-1.95284	0.00166463
ENSCAFG00000040537	5.93623	1.53857	-1.94796	0.00166463
ZSWIM4	3.02035	0.784465	-1.94493	0.00166463
COL6A1	60.8866	15.8359	-1.94292	0.00166463
METRNL	9.7525	2.53655	-1.9429	0.00166463
ENSCAFG00000048256	2.27051	0.590712	-1.94249	0.00166463
GATM	8.02386	2.08971	-1.94099	0.00166463
MT2A,MT3	122.366	31.8745	-1.94073	0.00166463
CEBPB	28.749	7.50504	-1.93758	0.00166463
ENSCAFG00000024864	0.958321	0.251004	-1.9328	0.0111445
TNS3	1.11965	0.293387	-1.93217	0.00166463
H2AJ	13.7264	3.60529	-1.92876	0.0052039
TNC	4.57633	1.20567	-1.92436	0.00166463
C1QTNF6	6.7637	1.78465	-1.92217	0.00166463
IRF8	0.928447	0.245201	-1.92086	0.0245705
DHX58	6.10863	1.61376	-1.92042	0.00166463
AKNA	0.87112	0.230811	-1.91616	0.00166463
SOX18	1.38926	0.368177	-1.91585	0.0304032

ARID5A	35.9921	9.54164	-1.91537	0.00166463
ENSCAFG00000043492	101.902	27.1699	-1.9071	0.00166463
TAGLN3	3.01304	0.803504	-1.90684	0.0135044
FBXO46	5.64515	1.50619	-1.9061	0.00166463
CDK1	24.9008	6.65354	-1.904	0.00166463
HMCN2	2.78462	0.746133	-1.89998	0.00166463
MAPK13	2.21903	0.595339	-1.89815	0.00414075
THBS4	32.5719	8.7495	-1.89636	0.00166463
BDNF	19.272	5.17713	-1.89628	0.00166463
FCER1G	63.8148	17.2168	-1.89007	0.00166463
ENSCAFG00000028473	9.8772	2.66934	-1.88762	0.00166463
ID3	33.4624	9.06721	-1.88381	0.00166463
NRDE2	6.22972	1.70647	-1.86815	0.0111445
COTL1	23.791	6.52923	-1.86543	0.00166463
ENSCAFG00000024733	3.41132	0.939001	-1.86113	0.0052039
NPPA	379.764	104.542	-1.86102	0.00414075
CD82	7.53258	2.0774	-1.85836	0.00166463
APLNR	5.55966	1.53347	-1.8582	0.00298108
COL4A2	107.406	29.6729	-1.85585	0.00166463
DUSP27	68.1855	18.8805	-1.85257	0.00166463
IL3RA	0.896868	0.248427	-1.85207	0.0240207
TRMT61A	3.31484	0.923036	-1.84448	0.00166463
TREM1	1.29842	0.36241	-1.84106	0.0471762
ENSCAFG00000047315	3.94615	1.10146	-1.84103	0.00166463
CHPF	10.0069	2.79417	-1.84051	0.00166463
NPNT	7.35189	2.07266	-1.82663	0.00166463
OLFML2B	15.1519	4.27287	-1.82622	0.00166463
GADD45B	97.3042	27.5128	-1.8224	0.00166463
CSPG4	5.30843	1.50309	-1.82036	0.00166463
LTF	0.998994	0.28333	-1.81799	0.0111445
OAF	14.182	4.02239	-1.81793	0.00166463
VAV1	1.86952	0.531853	-1.81357	0.00166463
ENSCAFG00000045499	6.55635	1.87019	-1.80971	0.00166463
MAP3K10	4.67743	1.33598	-1.80782	0.00298108
ENSCAFG00000040846	22.5424	6.44041	-1.80742	0.00166463
C1QC	19.8028	5.66322	-1.80601	0.00166463
ISLR	3.76817	1.08226	-1.79982	0.00166463
ENSCAFG00000028982	4.53239	1.30283	-1.79862	0.00166463
CPT1C	0.6525	0.18761	-1.79824	0.0111445
SLC37A2	1.8913	0.545357	-1.79411	0.00166463
MRPL57	52.2454	15.0824	-1.79244	0.0484275
PGF	5.90217	1.70417	-1.79218	0.00166463
CISH	62.3361	18.0272	-1.78989	0.00166463
FHL1	1101.44	319.19	-1.7869	0.00166463
JUND	13.1942	3.82913	-1.78482	0.00166463
EBF4	0.96159	0.279086	-1.78471	0.00709292
MATK	0.929972	0.271284	-1.77738	0.0209928
SLC7A5	3.21508	0.943837	-1.76824	0.00166463
PIK3R5	2.72986	0.80166	-1.76777	0.00166463
ALOX5AP	5.51628	1.62007	-1.76764	0.00618756
MROH5	10.4071	3.06146	-1.76528	0.00166463
ENSCAFG00000044488	15.2125	4.47866	-1.76412	0.00166463
ANKRD9	8.92364	2.63045	-1.76232	0.00166463

TRAF1	2.10425	0.620519	-1.76176	0.00298108
TSPO	20.2808	6.00244	-1.7565	0.0156032
ENSCAFG00000044303	3.42118	1.0127	-1.75629	0.0163086
VWCE	4.59029	1.36011	-1.75486	0.00166463
PLEC	67.8978	20.126	-1.75431	0.00166463
MEX3B	1.2185	0.362204	-1.75023	0.0356752
RITA1	85.6301	25.4682	-1.74942	0.00166463
FGF18	1.83231	0.545065	-1.74916	0.0348071
MXRA5	12.099	3.60008	-1.74879	0.00166463
MARCKSL1	21.5476	6.41838	-1.74725	0.0222118
TMEM88	17.1731	5.11671	-1.74687	0.00166463
TYRO3	1.10756	0.33039	-1.74514	0.00298108
FAM222A	1.83699	0.548573	-1.74359	0.00166463
CCN3	446.434	133.414	-1.74253	0.00166463
PIK3CD	1.15323	0.344659	-1.74244	0.00166463
DUSP8	12.31	3.68061	-1.74182	0.0170082
KLHL30	8.21187	2.45663	-1.74103	0.00166463
CD74	102.298	30.6515	-1.73875	0.00166463
XAF1	2.64954	0.794294	-1.738	0.00298108
CDC42EP1	8.62566	2.58888	-1.73631	0.00166463
NINJ1	7.09713	2.13134	-1.73547	0.00166463
CYP17A1	0.680918	0.20464	-1.73439	0.0142522
FASN	5.59986	1.68342	-1.734	0.00166463
CRIP1	227.312	68.4858	-1.7308	0.00166463
SPHK1	6.53862	1.97088	-1.73014	0.00166463
ENSCAFG00000000701	26.7505	8.06725	-1.72942	0.008824
C20H19orf71	3.38149	1.02058	-1.72827	0.00166463
TRAF3IP2	4.97313	1.50244	-1.72684	0.00166463
ACHE	1.97897	0.598519	-1.72528	0.00166463
FBRSL1	7.82049	2.36731	-1.72401	0.00166463
TGM2	490.703	148.607	-1.72335	0.00166463
LAMC3	2.42441	0.734446	-1.72291	0.00166463
DUSP13	1.65749	0.502501	-1.7218	0.0186537
CREB3L1	3.71073	1.12514	-1.7216	0.00166463
C1RL	1.31677	0.399829	-1.71955	0.00166463
DISP3	2.18775	0.66455	-1.719	0.00166463
CREB5	35.9901	10.9533	-1.71623	0.00166463
BPI	11.9879	3.64847	-1.71622	0.00166463
NOL3	6.41942	1.95696	-1.71382	0.00166463
IRX3	5.05689	1.54351	-1.71203	0.00166463
ADAMTS2	6.88804	2.10705	-1.70887	0.00166463
CLSTN3	2.35056	0.71918	-1.70858	0.008824
LMNB2	3.14629	0.96343	-1.7074	0.00166463
ENSCAFG00000006007	15.9503	4.88637	-1.70675	0.00166463
MRC2	8.24327	2.52699	-1.7058	0.00166463
DCHS1	4.79832	1.47104	-1.70569	0.00166463
ENSCAFG00000005852	3.96823	1.21692	-1.70526	0.00298108
ROBO4	11.3529	3.48282	-1.70473	0.00166463
MSC	3.10487	0.956361	-1.6989	0.00298108
COL4A1	160.415	49.5708	-1.69424	0.00166463
GMIP	1.89571	0.586708	-1.69202	0.00166463
ENSCAFG00000046373	25.8232	8.03929	-1.68353	0.00166463
PTAFR	0.898837	0.280187	-1.68167	0.0313973

RTL6	12.7747	3.99181	-1.67818	0.00166463
TEDC1	3.73332	1.16857	-1.67571	0.00166463
CEP131	20.1241	6.29989	-1.67553	0.00166463
ZNF513	4.99886	1.56891	-1.67184	0.00166463
FOXS1	6.7541	2.12599	-1.66763	0.00414075
MAMLD1	0.870396	0.27431	-1.66586	0.00166463
ENSCAFG00000049294	4.62952	1.46584	-1.65913	0.00414075
GRWD1	3.75045	1.18774	-1.65884	0.00166463
ADRA2A	0.852953	0.270461	-1.65705	0.0439079
ENDOV	2.12704	0.674699	-1.65653	0.00414075
FLNA	50.2922	15.9666	-1.65527	0.00166463
MAPK8IP1	0.892631	0.283407	-1.65519	0.0104658
ENSCAFG00000016577	8.06131	2.56473	-1.65221	0.0333266
CEP164	3.84922	1.22492	-1.65188	0.00166463
KCTD15	5.26049	1.68209	-1.64494	0.00166463
ENSCAFG00000049058	12.862	4.11408	-1.64448	0.00166463
ENSCAFG00000001686	5.97141	1.91145	-1.64341	0.011901
CADM3	3.23598	1.0359	-1.64332	0.00166463
PGAM2	470.124	150.517	-1.64311	0.00166463
ZNF219	8.10346	2.60136	-1.63927	0.00709292
DNAJB5	176.295	56.7263	-1.6359	0.00166463
BLVRB	8.10583	2.61703	-1.63103	0.00166463
TICAM1	4.97967	1.60781	-1.63096	0.00166463
SHB	20.9172	6.75577	-1.6305	0.00166463
DOK1	2.46238	0.795367	-1.63036	0.00414075
CDKN1A	40.7772	13.1819	-1.6292	0.00166463
CLEC3B	46.3362	15.0162	-1.62562	0.00166463
ACTN1	30.3411	9.83542	-1.62522	0.00166463
TNFRSF1B	13.4914	4.37431	-1.62492	0.00166463
DENND1C	0.774401	0.251201	-1.62424	0.008824
ARSA	7.74323	2.51647	-1.62154	0.00166463
P3H4	3.44936	1.12133	-1.62112	0.00166463
HLX	1.03005	0.336083	-1.61582	0.0156032
MROH1	7.02995	2.29409	-1.61559	0.0163086
FSCN1	15.4634	5.04623	-1.61558	0.00166463
MRTFA	7.06397	2.3061	-1.61502	0.00166463
KCNF1	4.23484	1.38274	-1.61477	0.00166463
EPHB2	0.862411	0.281614	-1.61466	0.00970753
TAGLN2	114.702	37.5127	-1.61245	0.00166463
DAPK3	121.869	39.8839	-1.61145	0.00166463
ARVCF	4.6001	1.50814	-1.60889	0.00166463
VPREB1	1.14751	0.37627	-1.60867	0.0468698
JUNB	62.6966	20.585	-1.60679	0.00166463
BOLA1	48.4692	15.9342	-1.60495	0.00166463
MYL5	26.8778	8.86257	-1.60062	0.00166463
ENSCAFG00000013668	2.00055	0.660224	-1.59937	0.00166463
GEM	4.72215	1.5585	-1.59929	0.00166463
TEAD4	27.2861	9.01677	-1.59748	0.00166463
ARHGEF40	5.49598	1.81698	-1.59683	0.00166463
C18H11orf95	2.36552	0.78298	-1.59511	0.0052039
CSPG5	1.5658	0.520624	-1.58859	0.011901
KIRREL1	1.57614	0.524528	-1.5873	0.00166463
LZTS1	1.77521	0.592231	-1.58376	0.00166463

CRABP2	3.91059	1.30905	-1.57887	0.0351794
FAM102A	7.89341	2.64612	-1.57677	0.00618756
RTP4	2.42396	0.812646	-1.57667	0.026814
RGS16	6.25087	2.09699	-1.57573	0.00166463
SYDE1	3.64037	1.23177	-1.56335	0.0439079
SH2B2	1.69842	0.57471	-1.56329	0.00298108
SPI1	4.03991	1.36924	-1.56094	0.00414075
TSTD1	36.4372	12.3731	-1.55821	0.0052039
GPC1	8.63902	2.93382	-1.55809	0.00166463
ENSCAFG00000049380	13.9184	4.7291	-1.55735	0.00298108
SLC6A6	41.1146	13.9873	-1.55554	0.00166463
ENSCAFG00000048027,PXN	32.0879	10.9196	-1.55511	0.00166463
CORO1A	2.83826	0.966542	-1.5541	0.00166463
COL1A2	63.7734	21.7366	-1.55282	0.00166463
FFAR2	6.10938	2.08234	-1.55282	0.00166463
THAP7	5.94314	2.03116	-1.54892	0.0209928
CACNA1H	19.105	6.5478	-1.54487	0.00166463
LRRC4	9.13921	3.13249	-1.54476	0.0111445
BIN2	1.34085	0.459602	-1.54469	0.00970753
PACSIN3	137.649	47.2356	-1.54304	0.00166463
MAP2K3	89.4982	30.7519	-1.54118	0.00166463
CACTIN	3.59121	1.23411	-1.541	0.00166463
ENSCAFG00000005768	7.3689	2.5332	-1.54049	0.00414075
RCC2	7.26551	2.49852	-1.53999	0.00166463
OSGIN1	3.93701	1.35858	-1.53501	0.0142522
TACC3	1.28882	0.444847	-1.53467	0.008824
LRMDA	0.715157	0.246854	-1.5346	0.008824
ENSCAFG00000017241	3.89966	1.3465	-1.53414	0.0240207
ENSCAFG00000019704	13.5859	4.69196	-1.53385	0.00166463
ENSCAFG00000030005	1.40589	0.485749	-1.5332	0.00166463
ENSCAFG00000031543	2.94341	1.0175	-1.53247	0.00166463
IGFBP3	3.11273	1.07665	-1.53164	0.00166463
ENSCAFG00000043502	935.144	323.454	-1.53163	0.00166463
RGMA	5.96691	2.06904	-1.52802	0.00166463
ETV7	0.915247	0.317455	-1.52761	0.00414075
ADGRA2	5.1479	1.78898	-1.52485	0.00166463
MID1IP1	12.6701	4.41253	-1.52175	0.00166463
ENSCAFG00000043167	4.4573	1.55308	-1.52104	0.00709292
CHRD	3.8784	1.35298	-1.51932	0.00166463
ALDH4A1	49.9696	17.4623	-1.51681	0.00166463
APOA1	140.177	49.0198	-1.51581	0.00166463
NCKAP5L	4.07022	1.42434	-1.51482	0.00166463
IL1RN	7.85663	2.7494	-1.51479	0.00166463
ENSCAFG00000049679,PEA15	303.459	106.268	-1.51379	0.008824
TIAM1	1.53341	0.537338	-1.51284	0.0104658
SCAND1	19.4122	6.80264	-1.51279	0.00166463
CFAP410	2.43109	0.852234	-1.51228	0.0288776
ENSCAFG00000020060	17.9209	6.29256	-1.50993	0.00970753
ZNF385A	1.56401	0.549695	-1.50855	0.00414075
PCDHGC3	17.3745	6.10908	-1.50795	0.00799923
NOC4L	3.59651	1.26528	-1.50714	0.0366339
CMTM3	6.7767	2.38411	-1.50713	0.00166463
PRELID1	26.5997	9.35863	-1.50704	0.00166463

MED25	14.3086	5.03468	-1.50691	0.00799923
FAS	6.42476	2.26118	-1.50656	0.00166463
ST6GALNAC4	58.0702	20.4711	-1.5042	0.00166463
ERRFI1	16.5248	5.83304	-1.50231	0.00166463
ZBTB47	45.9444	16.2432	-1.50005	0.00166463
ADAMTS15	5.09024	1.80096	-1.49896	0.00166463
ZNHIT2	2.86668	1.01615	-1.49626	0.0204062
DUSP8	18.1893	6.44889	-1.49596	0.00166463
C5H16orf74	14.1066	5.00268	-1.49559	0.0351794
CAPG	4.15222	1.47345	-1.49469	0.00166463
TNFSF12	7.8328	2.78081	-1.49402	0.0170082
HSPB1	3153.01	1120.14	-1.49306	0.00166463
ENSCAFG00000041107,POFUT2	6.92731	2.46156	-1.49272	0.00298108
TNXB	9.79429	3.48436	-1.49105	0.0431027
HAS1	1.34878	0.479888	-1.49089	0.0263476
ENSCAFG00000047466	1.55874	0.554602	-1.49086	0.0435493
TMSB10	279.894	99.5865	-1.49086	0.00166463
RCN3	6.93126	2.46855	-1.48945	0.00166463
STOML1	11.1602	3.98756	-1.48478	0.00166463
ENSCAFG00000041551	340.957	121.878	-1.48415	0.008824
NCDN	3.65568	1.30851	-1.48221	0.00166463
PTX3	26.9425	9.66027	-1.47975	0.00166463
RASSF5	3.04238	1.09112	-1.47939	0.00166463
BCAR1	30.8626	11.0689	-1.47935	0.00166463
AGRN	5.29566	1.89953	-1.47917	0.011901
IL27RA	3.08229	1.10674	-1.47769	0.00166463
MRPL54	15.0808	5.41613	-1.47738	0.00166463
COL5A3	15.6045	5.60787	-1.47644	0.00166463
BMP1	9.92447	3.56694	-1.47631	0.00166463
PLOD1	21.5431	7.74972	-1.47501	0.00166463
LZTS3	4.05398	1.4608	-1.47258	0.00166463
DDX54	15.6663	5.64651	-1.47224	0.0156032
SERPINE1	189.238	68.2542	-1.47121	0.00166463
IL18BP	15.0723	5.44455	-1.46902	0.00166463
COL15A1	47.1968	17.0654	-1.46761	0.00166463
GNAT1,SEMA3F	16.2982	5.89357	-1.4675	0.00166463
ITGA3	5.67162	2.05109	-1.46737	0.00166463
C5AR1	2.49948	0.904087	-1.46709	0.00709292
SDF2L1	5.30152	1.91805	-1.46677	0.0192697
AP5B1	1.2049	0.436782	-1.46393	0.0181454
GPIHBP1	10.5714	3.83338	-1.46347	0.0104658
EFNB1	3.35674	1.21766	-1.46295	0.00166463
ENSCAFG00000042538	1.89543	0.68772	-1.46263	0.0052039
MED15	35.4164	12.8506	-1.46258	0.00166463
PGGHG	1.12149	0.407541	-1.46039	0.0163086
SLC2A6	3.39878	1.23562	-1.45978	0.00166463
PRR32	11.3985	4.14397	-1.45976	0.00166463
SLA	1.10576	0.402043	-1.45962	0.0298794
SDC3	20.7992	7.56881	-1.45839	0.00166463
FBLN1	37.5013	13.6549	-1.45752	0.00166463
ENSCAFG00000030940	1.77287	0.645998	-1.45649	0.00166463
MEAK7	2.1368	0.778636	-1.45643	0.0142522
IP6K2	18.7187	6.82225	-1.45616	0.0181454

PLEKHO1	39.821	14.5676	-1.45076	0.00166463
ATXN7L2	1.50053	0.549133	-1.45025	0.0052039
ALOX5	1.18121	0.432303	-1.45015	0.0257323
SLC43A2	1.69326	0.620154	-1.4491	0.0186537
VIPR2	11.842	4.33884	-1.44853	0.00166463
PVR	53.3972	19.5659	-1.44842	0.00166463
MPEG1	4.88056	1.78895	-1.44793	0.00166463
SCRIB	15.2006	5.57752	-1.44644	0.00166463
SERPINE2	23.5937	8.66355	-1.44537	0.00166463
TSPAN15	25.1903	9.26699	-1.44269	0.00166463
RRAS	57.3917	21.1222	-1.44208	0.00166463
ENSCAFG00000016581	143.874	53.0155	-1.44032	0.00166463
MANEAL	2.16974	0.799598	-1.44018	0.00166463
DUSP10	6.97013	2.56914	-1.4399	0.00166463
ENSCAFG00000009988	929.344	342.669	-1.4394	0.00166463
EPHB6	5.74552	2.11893	-1.4391	0.00166463
PPP1R1A	28.489	10.5071	-1.43904	0.00166463
IRF5	1.3362	0.493018	-1.43842	0.0222118
TSPAN18	14.7643	5.45259	-1.4371	0.0475889
TGFBI	20.9003	7.72224	-1.43643	0.00166463
EML3	4.44422	1.64423	-1.43452	0.00166463
ZNF628	1.19304	0.442006	-1.4325	0.011901
ENSCAFG00000037581	1.78974	0.663414	-1.43177	0.0343735
LTBP3	12.0642	4.47262	-1.43154	0.00166463
PLXNB2	7.3465	2.72365	-1.43152	0.0480651
SDK2	5.12836	1.90714	-1.42709	0.00166463
MICALL2	1.89277	0.707241	-1.42023	0.0052039
NSMF	3.70113	1.38319	-1.41997	0.00166463
TNS2	24.3625	9.10933	-1.41925	0.00166463
ARHGAP30	1.96971	0.736734	-1.41877	0.00298108
PHC2	27.4848	10.2828	-1.4184	0.00166463
TBCC	27.1804	10.175	-1.41753	0.00166463
ENSCAFG00000005537	25.0286	9.38397	-1.41531	0.00166463
SRC	16.7699	6.28788	-1.41523	0.00166463
NEURL2	10.9075	4.09307	-1.41407	0.00799923
EPHA2	11.2005	4.209	-1.41201	0.00166463
GPX1	138.855	52.1871	-1.41181	0.00166463
LRP1	9.47688	3.56282	-1.41139	0.00166463
LIPE	14.5323	5.47395	-1.40861	0.00166463
DUSP2	2.46452	0.928629	-1.40813	0.0404086
C1QTNF1	17.4713	6.58791	-1.40709	0.0192697
DUSP6	17.3001	6.52636	-1.40643	0.00166463
ENSCAFG00000016245	1.58832	0.59926	-1.40625	0.00298108
SUSD2	5.4859	2.07025	-1.40592	0.00166463
SH3TC1	0.912462	0.344378	-1.40577	0.0142522
FBXL7	1.14704	0.433091	-1.40517	0.026814
DHRS3	14.9807	5.65644	-1.40514	0.00166463
POLRMT	3.91162	1.47751	-1.40459	0.00166463
ACTN4	85.6644	32.398	-1.40279	0.00166463
ENSCAFG00000041098	0.823199	0.311699	-1.40109	0.0298794
THRSP	5.98948	2.26946	-1.40008	0.0450444
ENSCAFG00000010209	1.37894	0.522542	-1.39994	0.0175929
SYK	0.92903	0.352406	-1.39849	0.0343735

SLC12A9	0.751755	0.285878	-1.39486	0.026814
CAPN15	1.8545	0.707457	-1.39032	0.00414075
PPP1R12C	19.533	7.45264	-1.39009	0.00166463
DLL1	1.50383	0.574551	-1.38813	0.0288776
PODN	7.58763	2.90001	-1.38759	0.00166463
HIC1	2.00088	0.76558	-1.38601	0.00166463
AKT1S1,PNKP	20.4833	7.8412	-1.3853	0.00166463
WSCD1	4.58704	1.75713	-1.38434	0.00166463
FMOD	5.84089	2.2376	-1.38423	0.00166463
NOD2	0.992876	0.380507	-1.38369	0.00298108
SBF1	13.3823	5.14125	-1.38014	0.0052039
ENSCAFG00000034748	5.09423	1.95723	-1.38005	0.0111445
FN1	54.2977	20.8767	-1.37899	0.00166463
TP53I11	45.1235	17.383	-1.3762	0.0181454
SLCO2B1	13.8333	5.3304	-1.37583	0.00166463
VDR	1.69007	0.651449	-1.37536	0.00166463
S1PR3	4.89058	1.88595	-1.37471	0.00166463
ANPEP	1.07519	0.41478	-1.37418	0.008824
CNN1	6.72904	2.59709	-1.37351	0.00298108
MICOS13	95.5394	36.8761	-1.37341	0.00166463
ENSCAFG00000031152	4.65371	1.79728	-1.37257	0.0293656
HSD3B7	30.5382	11.7977	-1.37211	0.0487642
OSBPL5	3.44833	1.33503	-1.36903	0.00414075
MRI1	3.76636	1.45824	-1.36895	0.0135044
ZNRF3	1.2132	0.469788	-1.36874	0.0163086
SHISA5	16.1019	6.23522	-1.36872	0.0186537
COMTD1	27.3731	10.6048	-1.36804	0.00166463
ENSCAFG00000013585	4.50403	1.74548	-1.36759	0.0351794
CYB5R3	226.142	87.7022	-1.36654	0.00166463
SHKBP1	4.26872	1.65665	-1.36554	0.00298108
ACOT7	3.10546	1.20561	-1.36505	0.0135044
MAN2C1	8.14991	3.16519	-1.36449	0.00166463
PML	13.0089	5.05602	-1.36342	0.00166463
NCKAP1L	1.35889	0.528803	-1.36163	0.008824
ENSCAFG00000000072	10.2061	3.97757	-1.35947	0.00298108
LAPTM5	8.474	3.30547	-1.35819	0.00166463
PMEPA1	11.5169	4.49252	-1.35816	0.00166463
NTN1	1.26446	0.493288	-1.35802	0.011901
PKD1	9.57767	3.73712	-1.35775	0.0186537
TLE3	5.22311	2.03811	-1.35768	0.00166463
SH3PXD2B	12.0538	4.70425	-1.35744	0.00166463
ENSCAFG00000048494	48.937	19.1326	-1.3549	0.00166463
UHRF1	0.845839	0.331164	-1.35284	0.0404086
POSTN	14.7582	5.78656	-1.35073	0.00166463
ADAMTS9	20.6369	8.09609	-1.34993	0.00166463
FBLN2	11.7217	4.60137	-1.34904	0.00166463
CCR1	1.2426	0.488005	-1.34839	0.0468698
PLEK	3.37906	1.32791	-1.34747	0.00166463
PSMB10	15.1201	5.94876	-1.34581	0.00799923
C12H6orf141	46.5991	18.3423	-1.34513	0.00166463
ENSCAFG00000032358	1.74271	0.686041	-1.34496	0.0454727
RRBP1	18.0148	7.09209	-1.3449	0.0298794
FOSL2	60.897	23.9916	-1.34384	0.00166463

MYOT	141.18	55.6619	-1.34277	0.00166463
THNSL2	15.6121	6.15914	-1.34187	0.0257323
UNC5B	7.92178	3.12729	-1.34091	0.00166463
SDK1	1.62909	0.64344	-1.34019	0.00166463
ITGBL1	9.58855	3.78844	-1.33971	0.00166463
FBLN5	39.6131	15.6637	-1.33856	0.00166463
FZD9	2.3162	0.915882	-1.33853	0.0216077
SRF	32.6661	12.9319	-1.33686	0.00166463
ENSCAFG00000041182	2.29379	0.90894	-1.33548	0.0343735
TRADD	3.14033	1.24533	-1.33439	0.0388821
CARD19	6.99215	2.77325	-1.33416	0.0156032
MIIP	3.29491	1.30684	-1.33415	0.0361288
EHD3	1.52876	0.606635	-1.33346	0.011901
NECAB3	1.43319	0.569234	-1.33214	0.011901
IDUA	7.84124	3.11463	-1.33202	0.0104658
BAMBI	14.1386	5.62355	-1.33009	0.0052039
GRAMD1A	12.356	4.91453	-1.33009	0.00166463
HOMER3	2.27517	0.90576	-1.32877	0.02735
RASA3	32.2413	12.8468	-1.3275	0.00166463
SLC6A17	2.89659	1.15459	-1.32698	0.00166463
ACKR1	3.48807	1.39066	-1.32666	0.00166463
CD44	42.4981	16.9554	-1.32565	0.00166463
ADAM19	20.9612	8.36698	-1.32495	0.00166463
ID1	14.6862	5.86282	-1.3248	0.00166463
MMP28	24.6738	9.85057	-1.3247	0.00166463
ENSCAFG00000029465	13.9799	5.58204	-1.32449	0.0328579
ENSCAFG00000039349	1.17027	0.467342	-1.3243	0.044634
ACTA1	15888.1	6346.03	-1.32402	0.0111445
SSC5D	1.77376	0.708954	-1.32305	0.00166463
SRPX2	4.79937	1.91878	-1.32266	0.00298108
TRAPPC5	62.4956	25.009	-1.32131	0.00166463
MYH10	41.0213	16.4201	-1.32091	0.00166463
SMIM1	14.0556	5.62629	-1.32089	0.011901
KCNN1	5.00422	2.00619	-1.31869	0.00166463
PDGFB	11.2653	4.51904	-1.3178	0.00298108
CCDC157	0.869965	0.34904	-1.31757	0.0487642
NLRC5	8.54043	3.43363	-1.31458	0.00166463
ENSCAFG00000005575	16.1303	6.48854	-1.31381	0.00166463
YBEY	5.99817	2.41418	-1.31299	0.00166463
MYBBP1A	13.7848	5.55151	-1.31213	0.00166463
SAMD11	1.09916	0.442704	-1.31198	0.0198195
SH3BGRL3	51.3046	20.681	-1.31078	0.00166463
DGKD	43.5596	17.5646	-1.31032	0.00166463
MGAT5B	2.33841	0.943126	-1.31001	0.0052039
IKBKE	8.13663	3.28235	-1.3097	0.00166463
CD320	6.65924	2.68927	-1.30814	0.0104658
COL6A3	34.3907	13.8888	-1.3081	0.00166463
ARMC6	3.81219	1.53994	-1.30775	0.00414075
HSPB6	2693.07	1087.98	-1.3076	0.00166463
PLEKHA7	4.75814	1.92243	-1.30747	0.00166463
BOP1	11.1993	4.52735	-1.30667	0.00166463
CPLX2,HRH2	2.23637	0.904938	-1.30527	0.0052039
INHBE	21.0863	8.53296	-1.30519	0.00166463

AVIL	1.49081	0.604021	-1.30343	0.011901
FLNB	16.7668	6.79414	-1.30324	0.00166463
ADM	30.7327	12.4551	-1.30304	0.00166463
ENSCAFG00000012367	9.04917	3.66965	-1.30214	0.00166463
PYCR3	2.90575	1.17843	-1.30205	0.0175929
ENSCAFG00000014991	23.3945	9.49064	-1.3016	0.00166463
MMP2	22.0856	8.96237	-1.30115	0.00166463
FTL	23.7429	9.63745	-1.30077	0.00166463
MAPK7	1.18467	0.480948	-1.30054	0.0293656
SLC25A23	5.36285	2.17888	-1.29941	0.00166463
ELK1	16.8172	6.83802	-1.29829	0.00166463
VPS37B	13.5523	5.51281	-1.29768	0.00166463
RND3	46.947	19.0972	-1.29767	0.00166463
ZDHHC8	8.83602	3.59502	-1.2974	0.00166463
CRIP2	214.857	87.4325	-1.29713	0.00166463
NOL6	4.21574	1.71591	-1.29681	0.00166463
ZFPL1	7.58217	3.08831	-1.29579	0.00414075
IBA57	4.55476	1.85681	-1.29455	0.00298108
PLAAT4	45.2277	18.4426	-1.29416	0.00298108
SIPA1L3	2.9747	1.21309	-1.29406	0.00166463
COL8A1	17.5812	7.17859	-1.29226	0.00166463
HLA-DQB1	13.2068	5.39259	-1.29223	0.0111445
PLIN3	15.9685	6.5207	-1.29213	0.00166463
RNPEPL1	16.7183	6.83537	-1.29034	0.00166463
TGFB2	9.41672	3.85014	-1.29031	0.00298108
BACH2	1.92715	0.788056	-1.2901	0.00414075
SERPINA3	3.50196	1.43228	-1.28985	0.0175929
CSF2RB	3.26971	1.33797	-1.28912	0.00166463
ABHD17A	27.4355	11.2281	-1.28893	0.00166463
INSYN1	16.2778	6.66214	-1.28885	0.00166463
SRMS	2.23845	0.916689	-1.288	0.0263476
EXD2	3.34439	1.37169	-1.28579	0.00709292
BAG3	159.039	65.2463	-1.28542	0.00166463
METTL1	10.3748	4.25672	-1.28527	0.00709292
FAM167B	24.6042	10.0993	-1.28465	0.00166463
ENSCAFG00000018753	222.188	91.2328	-1.28416	0.00166463
PLXNA4	4.05955	1.66689	-1.28416	0.00166463
TCF21	4.39058	1.80326	-1.2838	0.008824
HCFC1R1	90.6328	37.258	-1.28248	0.008824
CSKMT,UQCC3	14.5182	5.96888	-1.28233	0.0425903
BTBD2	21.312	8.76249	-1.28225	0.00166463
RPL37A	916.664	377.105	-1.28143	0.00166463
LSP1	54.5154	22.4308	-1.28118	0.00166463
SEMA6B	1.45347	0.598656	-1.2797	0.0245705
SIRT6	1.70145	0.700797	-1.2797	0.0464503
LRRC31	1.17391	0.483753	-1.27898	0.0379891
PRPH	4.47069	1.84393	-1.27771	0.00799923
TENT5B	4.31245	1.77982	-1.27678	0.00298108
ZNF579	4.78473	1.9751	-1.27651	0.00166463
CSRP3	3243.58	1339.44	-1.27596	0.00166463
GSN	159.478	65.8825	-1.27539	0.00166463
ADAMTS8	19.8362	8.1972	-1.27493	0.00166463
TESPA1	2.15354	0.889969	-1.27488	0.0175929

ENSCAFG00000025115	1.6418	0.678688	-1.27446	0.0204062
HK2	9.69444	4.0077	-1.27438	0.00166463
DLGAP4	20.9065	8.64638	-1.27379	0.00166463
TSSC4	9.29665	3.8508	-1.27155	0.00298108
ENSCAFG00000044872	104.736	43.3872	-1.27141	0.00166463
ASMTL	4.75596	1.97042	-1.27124	0.011901
FGD2	1.0083	0.417941	-1.27055	0.0398565
NPAS2	7.27724	3.01723	-1.27017	0.00166463
CSRP1	95.3521	39.554	-1.26944	0.00166463
TNIP3	3.8623	1.60336	-1.26836	0.0111445
AKAP12	14.3176	5.94616	-1.26776	0.00166463
CSNK1G2	10.5107	4.36556	-1.26762	0.00166463
NUDT14	6.77285	2.81335	-1.26747	0.0356752
ARHGDIG,PDIA2	25.3469	10.5335	-1.26682	0.0127236
ENSCAFG00000043545	3.48709	1.45057	-1.2654	0.0370398
KLHL40	81.7061	34.0181	-1.26414	0.00166463
HRAS	13.939	5.80742	-1.26316	0.00298108
GATAD2A	16.5011	6.87709	-1.26269	0.00166463
MCAM	35.8652	14.9532	-1.26213	0.00709292
ROR1	2.2215	0.926264	-1.26204	0.0198195
GPRC5C	10.8062	4.51091	-1.26037	0.00166463
SERPINH1	88.3682	36.9032	-1.25978	0.00166463
MAP7D1	172.694	72.1397	-1.25935	0.00166463
DES	4706.53	1966.88	-1.25875	0.00414075
IL6	14.1681	5.92985	-1.25658	0.00166463
PREX1	2.3128	0.968201	-1.25626	0.00166463
PXDN	12.9681	5.43482	-1.25467	0.00166463
FKBP11	7.2033	3.01971	-1.25425	0.0454727
PFKFB3	10.0235	4.20222	-1.25415	0.00166463
PLEKHM2	85.5377	35.8742	-1.25361	0.00166463
SLC29A4	14.2483	5.98059	-1.25243	0.00166463
PAPPA	1.75363	0.736424	-1.25173	0.00414075
RELA	48.2814	20.2982	-1.25012	0.0111445
ARHGAP23	7.80043	3.28819	-1.24626	0.00166463
NID2	33.2088	14.0004	-1.24609	0.008824
ANKHD1	70.8526	29.8808	-1.2456	0.0491872
ENSCAFG00000031869	19.6354	8.28364	-1.24512	0.00709292
CYGB	11.1117	4.69163	-1.24391	0.0471762
THBS1	109.44	46.2105	-1.24384	0.00166463
OAS3	3.00601	1.26982	-1.24323	0.0052039
RCAN1	374.55	158.226	-1.24317	0.00166463
MICAL1	1.08061	0.456518	-1.2431	0.00298108
MPRIP	200.371	84.6694	-1.24276	0.00166463
ALPK3,ZNF592	66.2931	28.0429	-1.24122	0.00166463
ITIH5	19.6113	8.29772	-1.2409	0.00166463
IFFO2	1.66373	0.70421	-1.24034	0.0251707
ENSCAFG00000013015	9.97125	4.22064	-1.24031	0.00166463
NOTCH4	10.9226	4.62933	-1.23845	0.00166463
TAF1C	2.20217	0.933868	-1.23764	0.00799923
HBEGF	40.9047	17.3594	-1.23655	0.00166463
PTK7	1.49853	0.636807	-1.23462	0.0104658
MAFF	35.8336	15.241	-1.23335	0.00166463
ECM1	33.364	14.1911	-1.23331	0.00166463

DMPK	66.6909	28.3743	-1.23291	0.00166463
PDLIM2	0.748444	0.318766	-1.2314	0.0454727
IRAK1	4.52474	1.92783	-1.23085	0.00414075
FZD1	3.66902	1.5645	-1.22969	0.00166463
D2HGDH	11.7989	5.03476	-1.22866	0.0251707
NACC1	5.78564	2.47234	-1.2266	0.00414075
ENC1	3.61406	1.54544	-1.2256	0.0175929
CMKLR1	4.18001	1.78791	-1.22524	0.00166463
TNIP2	3.5676	1.52603	-1.22517	0.0111445
PDLIM3	70.3851	30.1112	-1.22497	0.00166463
MGP	337.289	144.384	-1.22407	0.00166463
P2RX3	1.51521	0.648795	-1.22368	0.0156032
TMUB1	5.73039	2.45377	-1.22363	0.00414075
CCND3	6.44857	2.76645	-1.22094	0.00799923
MIER2	6.24638	2.68021	-1.22068	0.00166463
TRIB1	42.0505	18.0448	-1.22053	0.00166463
TKT	34.901	14.9856	-1.21968	0.00166463
TSC22D2	32.5498	13.9927	-1.21798	0.00618756
WDR4	5.94691	2.55765	-1.21732	0.00298108
BTG2	146.695	63.1708	-1.21549	0.00166463
SLC52A1	5.86078	2.52424	-1.21524	0.00799923
CD40	56.7043	24.4232	-1.2152	0.00618756
FAAP100	1.83544	0.791217	-1.21398	0.0135044
ENSCAFG00000042931	13.6255	5.87989	-1.21245	0.00298108
SCAF1	15.7281	6.78727	-1.21244	0.00166463
ENSCAFG00000008503	4.08884	1.76508	-1.21196	0.026814
SPINDOC	6.62303	2.86098	-1.21098	0.00618756
VASH1	33.2156	14.3513	-1.21068	0.00166463
TMEM189	39.681	17.1464	-1.21054	0.00166463
ENSCAFG00000013154	2.17341	0.940238	-1.20887	0.0375549
FNDC1	1.42024	0.615039	-1.20739	0.00414075
ELF4	1.19447	0.517527	-1.20666	0.0156032
B4GALT2	11.1125	4.81764	-1.20578	0.00414075
CYP46A1	2.65387	1.15068	-1.20562	0.00414075
C1R	21.5996	9.37058	-1.20479	0.00166463
ENSCAFG00000037819	21.1724	9.18747	-1.20444	0.00166463
SHMT2	4.18963	1.81958	-1.20322	0.0111445
ENSCAFG00000029582,ENSCAFG00000030509	1.05032	0.456181	-1.20315	0.0421371
IL4R	15.7462	6.84384	-1.20213	0.0370398
LOXL1	11.2484	4.88911	-1.20207	0.00166463
AMPD2	8.24327	3.58532	-1.20111	0.00166463
DLA-12	70.1283	30.5017	-1.20111	0.0052039
ENSCAFG00000038281	1.1615	0.505365	-1.20059	0.0480651
RASL10B	25.8142	11.2336	-1.20034	0.00166463
SMUG1	8.64164	3.76068	-1.20031	0.0163086
ENSCAFG00000031575	3.75374	1.63463	-1.19936	0.0052039
SPARC	411.859	179.364	-1.19926	0.00166463
MKNK2	18.9516	8.26054	-1.19801	0.00166463
EXOSC4,GPAA1	25.5263	11.1285	-1.19772	0.00298108
ITGA7	91.3905	39.8975	-1.19575	0.00166463
CASKIN2	9.88663	4.31815	-1.19507	0.0251707
PPRC1	12.1087	5.2976	-1.19264	0.00166463
ENSCAFG00000042539	4.60537	2.0158	-1.19196	0.0186537

MYH9	101.004	44.2633	-1.19023	0.0104658
GPRC5A	2.42922	1.065	-1.18964	0.0319719
RNASEK	73.0159	32.0111	-1.18964	0.00166463
TSKU	3.34845	1.46864	-1.18901	0.00166463
ATN1	72.3649	31.7713	-1.18757	0.00166463
CIC	6.89317	3.02722	-1.18717	0.0216077
AKT1	63.1613	27.7646	-1.1858	0.00166463
SLC9A3R1	1.86782	0.821482	-1.18505	0.026814
SNAI2	9.37207	4.12425	-1.18424	0.008824
CTSC	10.5115	4.62653	-1.18397	0.00166463
ENSCAFG00000002916	8.84121	3.89162	-1.18387	0.00166463
CD209	25.4134	11.1906	-1.1833	0.00298108
KIF23,PAQR5	7.97979	3.51486	-1.18288	0.00618756
ULK1	24.0237	10.5863	-1.18225	0.00166463
DMAP1	92.7205	40.8766	-1.18161	0.00166463
CD248	5.99511	2.6438	-1.18118	0.00166463
PRRC2A	40.8836	18.0297	-1.18115	0.00166463
GRB10	11.58	5.10818	-1.18075	0.00166463
SCRN2	3.12435	1.37825	-1.18072	0.0209928
ENSCAFG00000041271	141.109	62.2763	-1.18006	0.0257323
EPHX3,NOTCH3	4.34526	1.91894	-1.17913	0.00166463
UBA7	1.32702	0.586074	-1.17903	0.0240207
ZNF500	2.11155	0.932585	-1.17899	0.0142522
VPS26C	13.1695	5.82266	-1.17745	0.00166463
ENSCAFG00000018333	9.19104	4.06635	-1.17649	0.00166463
ENSCAFG00000003818	158.009	69.9131	-1.17638	0.00166463
MALL	5.52847	2.44739	-1.17564	0.0484275
TRABD2B	1.54824	0.685397	-1.17562	0.0487642
DPYSL3	8.13478	3.60507	-1.17408	0.00166463
TSHZ2	3.6885	1.63468	-1.17402	0.00298108
BCAM	76.6055	33.9516	-1.17397	0.0450444
ENSCAFG00000023394	24.3433	10.7929	-1.17344	0.00166463
GFPT2	24.3798	10.8142	-1.17276	0.00166463
MYL4	197.921	87.9098	-1.17083	0.0319719
KLHL34	11.9471	5.30718	-1.17064	0.00414075
ADGRD1	2.98418	1.32573	-1.17055	0.0313973
ARHGAP1	25.1706	11.1878	-1.16982	0.00166463
KLF5	14.4218	6.41111	-1.16961	0.00166463
ENSCAFG00000049841	10.6139	4.71875	-1.16947	0.008824
SHROOM3	4.57857	2.03636	-1.16891	0.00166463
THY1	17.4426	7.75907	-1.16866	0.00166463
SLC22A17	4.95144	2.20415	-1.16763	0.00618756
NAGLU	1.80733	0.804623	-1.16747	0.02735
EXOC3L1	2.09196	0.931768	-1.16681	0.00414075
TUBB2A	6.82396	3.04014	-1.16647	0.00970753
A4GALT	13.3711	5.9623	-1.16517	0.00970753
B3GAT3	16.0566	7.16824	-1.16347	0.00298108
IRF2BP1	2.84222	1.269	-1.16332	0.0484275
CERCAM	14.8933	6.64972	-1.1633	0.00166463
ENSCAFG00000028930	65.5734	29.2842	-1.16299	0.0104658
CAPN10	2.94375	1.31548	-1.16207	0.0277976
DUS1L	18.857	8.42712	-1.16199	0.00166463
DOT1L	10.046	4.4915	-1.16135	0.00166463

MXRA8	2.69306	1.20437	-1.16097	0.0313973
CD151	156.11	69.8558	-1.16011	0.00166463
RPL8	363.143	162.499	-1.16011	0.00166463
COL5A2	30.3133	13.5657	-1.15999	0.00166463
HCLS1	13.259	5.93484	-1.15969	0.00166463
ENSCAFG00000029967	20.8934	9.3557	-1.15913	0.0404086
ENSCAFG00000028453	3.26462	1.46203	-1.15894	0.00414075
RGS2	9.47277	4.24272	-1.1588	0.0104658
DRAM1	4.75855	2.13149	-1.15866	0.0111445
ACTB	71.4083	32.0277	-1.15677	0.00166463
ADORA2A	1.93743	0.869217	-1.15635	0.0251707
EMP1	119.116	53.4465	-1.1562	0.0379891
BMP4	4.16149	1.8686	-1.15515	0.008824
NOTCH1	5.98226	2.68666	-1.15488	0.00166463
TAGLN	103.985	46.7027	-1.15479	0.00618756
BCL9L	5.32838	2.39569	-1.15326	0.00166463
EIPR1	8.64856	3.88904	-1.15304	0.00166463
ENSCAFG00000017257	22.933	10.3127	-1.153	0.00166463
SLC39A13	6.00698	2.7023	-1.15246	0.0163086
NPEPL1	9.65174	4.3426	-1.15223	0.00166463
DNAJB1	29.873	13.4444	-1.15184	0.00166463
PDIA4	66.8884	30.112	-1.15142	0.00166463
TRIM8	13.4096	6.03698	-1.15137	0.0351794
HYOU1	16.1921	7.29029	-1.15124	0.00166463
RGL1	12.1502	5.47049	-1.15124	0.00166463
CDH8	7.32705	3.31067	-1.14611	0.00166463
ETS1	44.7598	20.2412	-1.14491	0.0351794
FAM98C	7.20867	3.26443	-1.1429	0.0333266
TLNRD1	5.39695	2.4443	-1.14272	0.0111445
GAS2L1	3.24745	1.47091	-1.14259	0.00414075
TMEM134	13.2026	5.98335	-1.14179	0.0104658
ENSCAFG00000010687	6.98084	3.16443	-1.14145	0.00166463
TMEM51	9.57756	4.34154	-1.14145	0.00166463
AEN	7.05802	3.20141	-1.14056	0.00166463
TESC	28.338	12.8572	-1.14016	0.00166463
ENSCAFG00000032238	7.34703	3.33362	-1.14007	0.00298108
RPS21	718.748	326.367	-1.13899	0.0356752
PLXNA3	1.43226	0.650652	-1.13833	0.0111445
CLDN4	11.6993	5.31498	-1.13828	0.00166463
ITGB3	4.67089	2.12421	-1.13677	0.00799923
DTX2	4.01486	1.82618	-1.13652	0.00799923
PHOSPHO1	11.0254	5.01751	-1.13579	0.00166463
FNDC11	4.45135	2.02577	-1.13577	0.0198195
EPN1	28.1019	12.7922	-1.1354	0.00166463
LMCD1	72.8136	33.2117	-1.13252	0.00166463
C27H12orf10	34.3486	15.6759	-1.13171	0.00166463
ENSCAFG00000029292	5.91542	2.70324	-1.12979	0.008824
DDN	4.05117	1.85207	-1.1292	0.00799923
AFAP1	5.21315	2.38407	-1.12873	0.00970753
AUTS2	12.1125	5.54053	-1.1284	0.0240207
MYO1C	62.0225	28.3804	-1.12789	0.00166463
HSPB7	1412.58	646.613	-1.12736	0.00166463
INPP5J	2.00649	0.920124	-1.12478	0.0127236

ARFGAP1	17.8381	8.18062	-1.12468	0.00166463
ENSCAFG00000030466	16.1833	7.42339	-1.12436	0.00166463
ZMIZ2	40.4809	18.5749	-1.12389	0.00166463
GTPBP3	2.47191	1.1348	-1.12319	0.0333266
ENAH	147.768	67.8846	-1.12217	0.00166463
ARHGEF15	9.64937	4.43476	-1.12158	0.00166463
SERTAD1	11.7238	5.39444	-1.11989	0.00799923
FXYD6	20.623	9.48953	-1.11985	0.00166463
C18H11orf24	8.19199	3.77009	-1.11961	0.00414075
ASNS	2.54915	1.17403	-1.11855	0.0408367
FES	8.28021	3.81414	-1.11831	0.00166463
GDPD5	1.96455	0.905245	-1.11782	0.044199
TTYH3	4.53885	2.09231	-1.11723	0.00414075
ITPK1	11.9753	5.52082	-1.11711	0.00166463
KLHL21	28.6417	13.2064	-1.11688	0.00166463
PACS2	27.3648	12.6245	-1.11609	0.00166463
ALKBH6	5.8667	2.70782	-1.11542	0.0135044
FLT3LG	5.21947	2.41176	-1.11382	0.0366339
MMP14	7.29472	3.37362	-1.11255	0.00166463
FAM160A2	5.81184	2.68794	-1.11249	0.00298108
ENSCAFG00000010596	27.3456	12.6497	-1.1122	0.00166463
CLTB,RNF44	102.003	47.2206	-1.11113	0.00298108
RRP12	11.6984	5.41671	-1.11082	0.00166463
AHDC1	5.53842	2.56483	-1.11061	0.00166463
ELOVL1	9.2774	4.2969	-1.11042	0.026814
TMEM8A	3.98307	1.84495	-1.1103	0.008824
SORBS3	83.8069	38.8209	-1.11023	0.00166463
ANKRD13D	4.00776	1.85772	-1.10926	0.0142522
TNRC18	4.85307	2.25135	-1.10811	0.00166463
SMOC2	20.276	9.40864	-1.10771	0.00166463
SORCS2	1.33604	0.620054	-1.10749	0.0052039
JPT1	28.7091	13.3326	-1.10655	0.00414075
NCLN	9.87911	4.58802	-1.10651	0.00166463
RUSC2	19.4472	9.03171	-1.10649	0.00166463
ILK	86.1719	40.021	-1.10646	0.00166463
HSF1	30.6652	14.2559	-1.10505	0.0052039
FAM78B	5.14144	2.39274	-1.10351	0.0425903
ENSCAFG00000018511	4.25117	1.97862	-1.10336	0.0416803
TGFB1I1	10.686	4.97663	-1.10249	0.0052039
ENSCAFG00000019779	4.43559	2.06711	-1.10151	0.039381
ARNTL	3.82851	1.78517	-1.10072	0.0222118
VARS1	12.5831	5.86824	-1.10048	0.00166463
CPSF1	10.0295	4.67771	-1.10038	0.00166463
RNF213	7.31637	3.41364	-1.09982	0.00414075
RPS6KA1	1.77497	0.828217	-1.09971	0.0328579
CDC42EP3	99.4858	46.4305	-1.09942	0.00166463
CRISPLD2	13.6549	6.37282	-1.09941	0.00166463
ENSCAFG00000042414,MAST4	19.8235	9.25195	-1.09938	0.00166463
APRT	14.9982	7.00552	-1.09822	0.0198195
VWA7	6.47005	3.02284	-1.09787	0.00298108
PPP1R35	7.23941	3.38335	-1.09742	0.0439079
FXYD1	681.012	318.393	-1.09687	0.0348071
KLF6	68.444	32.018	-1.09604	0.00166463

HCFC1	8.41745	3.93984	-1.09525	0.00166463
TNKS1BP1	8.54241	3.99889	-1.09505	0.00166463
LLGL1	2.40808	1.12728	-1.09504	0.0181454
SLC19A1	2.37238	1.11124	-1.09417	0.0388821
RHBDD3	6.52446	3.05673	-1.09387	0.00799923
IFRD2	10.5644	4.94974	-1.09379	0.0484275
EVI5L	8.4528	3.96123	-1.09348	0.00166463
SRM	10.5912	4.96401	-1.09329	0.00709292
BTBD6	22.8481	10.7101	-1.09311	0.0263476
ABCC10	2.41948	1.13438	-1.09279	0.00298108
GRIK3	1.32155	0.61963	-1.09276	0.0127236
FAP	10.4006	4.88204	-1.0911	0.00298108
ENSCAFG00000005629	8.06962	3.79477	-1.08849	0.00618756
EFEMP2	6.4923	3.05333	-1.08835	0.0170082
PPM1M	4.62755	2.17665	-1.08814	0.0209928
ZNF358	14.3275	6.73974	-1.08802	0.00166463
TCAP	3374.8	1588.24	-1.08737	0.0480651
ZSWIM8	17.1848	8.08759	-1.08736	0.00166463
CCL7	364.736	171.926	-1.08506	0.00166463
PHF2	6.66543	3.14381	-1.08418	0.00166463
AMOTL2	21.248	10.0272	-1.08341	0.00166463
BCL6,RTP2	40.4208	19.0794	-1.08308	0.00166463
DLL4	6.37923	3.01122	-1.08304	0.00166463
ADGRE1	2.58148	1.21872	-1.08284	0.0263476
ENSCAFG00000044026	185.063	87.3874	-1.08252	0.00166463
RELL1	26.4405	12.4946	-1.08145	0.0142522
PCNX2	0.924073	0.436895	-1.08072	0.0408367
SSBP4	8.76637	4.14537	-1.08048	0.00709292
ZNF516	3.63856	1.72096	-1.08016	0.00166463
ADAM15,DCST1	10.028	4.74422	-1.07979	0.0216077
ENSCAFG00000042378	122.163	57.8064	-1.07951	0.00166463
REXO4	38.0811	18.0245	-1.07912	0.00166463
RHOD	41.0809	19.446	-1.07899	0.0104658
RNPEP	7.37336	3.49111	-1.07864	0.0209928
ETV5	1.23097	0.582844	-1.07862	0.044199
GCAT	6.59755	3.1268	-1.07724	0.011901
CHST3	6.61578	3.1359	-1.07703	0.00166463
SPEG	24.5497	11.638	-1.07686	0.00166463
ABL1	34.6956	16.4479	-1.07685	0.00166463
PEMT	15.73	7.46391	-1.07551	0.00618756
PPP1R37	13.5315	6.42346	-1.0749	0.00166463
NKX2-5	13.958	6.63553	-1.07281	0.0111445
PPP1R9B	41.8629	19.9189	-1.07154	0.00166463
ENSCAFG00000030770	5.53693	2.63577	-1.07086	0.00166463
DBN1	5.22636	2.48961	-1.06989	0.00970753
ADGRE5	8.51133	4.05536	-1.06956	0.008824
ENSCAFG00000010718	5.03791	2.40107	-1.06915	0.0348071
MMD	35.0283	16.6973	-1.06891	0.00709292
RHBDF1	14.6439	6.98554	-1.06786	0.00166463
PPP1R18	10.6881	5.10099	-1.06716	0.00166463
PFKL	23.2621	11.1236	-1.06435	0.00166463
ZDHHC1	14.849	7.10186	-1.0641	0.00618756
FOXK1	7.48266	3.57969	-1.06371	0.00618756

RNF157	12.5485	6.00444	-1.06341	0.00166463
LGI3	4.55406	2.18189	-1.06157	0.00799923
RPS26	348.144	166.828	-1.06132	0.00166463
CRYAB	5673.75	2720.97	-1.06018	0.00166463
SLC2A4RG	13.6277	6.53871	-1.05947	0.00166463
IKZF1	2.07043	0.993747	-1.05898	0.0150001
ADRA1B	26.481	12.7108	-1.0589	0.00298108
JPH1	14.1811	6.8077	-1.05873	0.00414075
STAT3	83.7101	40.1958	-1.05836	0.00166463
MTA1	9.0141	4.32874	-1.05824	0.00618756
PHLDB1	38.111	18.3121	-1.05741	0.00298108
LAMC2	4.21699	2.02718	-1.05674	0.00618756
SNX15	13.17	6.33121	-1.05671	0.0104658
PLOD3	18.7454	9.0133	-1.05641	0.00166463
PIEZO1	2.7799	1.33764	-1.05534	0.00414075
TBX2	2.02259	0.973343	-1.05518	0.0356752
JAG2	3.44682	1.65913	-1.05484	0.008824
HGH1	3.86305	1.85986	-1.05455	0.0192697
XXYLT1	9.47633	4.56488	-1.05375	0.0052039
FBN1	41.726	20.1003	-1.05373	0.00166463
RBM10	17.2308	8.30357	-1.05319	0.00166463
ZDHHC9	22.589	10.8861	-1.05313	0.011901
GJC2,GUK1	29.0956	14.0298	-1.05231	0.0351794
RASSF2	4.78781	2.30898	-1.05211	0.0150001
PRC1	1.71117	0.82577	-1.05118	0.0375549
SH3GL1	16.5826	8.00347	-1.05097	0.00166463
NRBP2	11.1096	5.36235	-1.05087	0.00414075
ZNRD2	13.6539	6.59088	-1.05077	0.0181454
PDE4B	15.9372	7.69344	-1.0507	0.00709292
ALG12	3.6196	1.74774	-1.05034	0.0398565
OPLAH	8.43555	4.07629	-1.04923	0.00166463
ENSCAFG00000049746	13.7208	6.63503	-1.04819	0.0351794
GNAI2	85.7951	41.4986	-1.04783	0.00166463
SLC1A5	9.58169	4.63626	-1.04732	0.011901
C8H14orf180	49.5223	23.9662	-1.04708	0.00166463
GAA	21.856	10.5842	-1.04612	0.00166463
P3H2	3.21443	1.55853	-1.04437	0.0181454
RAI14	8.0739	3.91909	-1.04275	0.008824
SF3B5	99.4543	48.2783	-1.04266	0.0343735
ST6GAL1	7.17331	3.48318	-1.04224	0.00166463
JPT2	18.4329	8.95105	-1.04215	0.0484275
ANTXR1	18.5551	9.03073	-1.0389	0.00166463
PAG1	2.60853	1.26963	-1.03884	0.0304032
FMNL3	20.9978	10.2233	-1.03838	0.00166463
MAST3	1.36589	0.665228	-1.03792	0.0204062
DCXR	35.41	17.2492	-1.03763	0.00799923
ZNF462	19.8662	9.68023	-1.03721	0.00166463
REXO1	6.13256	2.98928	-1.03669	0.00414075
PAK4	5.49516	2.67884	-1.03655	0.0127236
C9H9orf16	87.8471	42.8763	-1.03481	0.00414075
WT1	5.20452	2.54039	-1.03471	0.030955
THOP1	8.20121	4.00601	-1.03367	0.00799923
GABBR2	1.13978	0.55743	-1.03189	0.0181454

CHERP	6.50781	3.18509	-1.03084	0.0170082
VCAN	23.1641	11.341	-1.03034	0.00166463
SEPTIN5	29.4482	14.4212	-1.02998	0.00166463
ENSCAFG00000020296	71.2675	34.9171	-1.02931	0.00799923
SPATS2L	5.81932	2.85348	-1.02813	0.0375549
ZBTB46	2.55625	1.25397	-1.02753	0.0361288
RAMP1	117.476	57.6611	-1.02669	0.00298108
MGAT4B	16.8471	8.272	-1.02619	0.00166463
TFPT	256.262	125.845	-1.02597	0.0484275
TRMT1	4.58897	2.2542	-1.02556	0.0313973
METTL26	21.1618	10.3955	-1.02551	0.026814
XYLT1	1.46197	0.718174	-1.02551	0.0135044
PCED1A	6.2151	3.05517	-1.02453	0.0228621
PGLS	19.3184	9.49877	-1.02416	0.00799923
ATP13A2	1.94093	0.955107	-1.02302	0.0348071
PRELP	19.3827	9.53984	-1.02273	0.00166463
RPS8	1437.55	708.023	-1.02174	0.0245705
EGFL7	10.7461	5.29925	-1.01996	0.008824
IFT43	13.5071	6.66371	-1.01932	0.0175929
KDM6B	8.5985	4.2427	-1.0191	0.00166463
NME1	13.5223	6.67332	-1.01887	0.0288776
CRTC1	2.6265	1.29705	-1.01791	0.0468698
SNX33	4.27612	2.11248	-1.01736	0.0135044
HDAC11	8.16721	4.03657	-1.01671	0.0209928
CDC42EP2	12.7133	6.28411	-1.01656	0.00166463
SEC61A1	23.1991	11.4705	-1.01614	0.00166463
ENSCAFG00000024644	5.07807	2.51125	-1.01587	0.0222118
CTSH	10.8579	5.3697	-1.01584	0.0186537
SERPINF1	108.059	53.4467	-1.01564	0.0111445
MLST8	8.07737	3.99552	-1.0155	0.0245705
PPFIA4	8.61583	4.2629	-1.01515	0.00298108
MARCHF3	34.4817	17.0678	-1.01456	0.00166463
AAMP	28.9894	14.3523	-1.01425	0.00166463
ENSCAFG00000049820	2.52686	1.25229	-1.01278	0.0288776
NEK6	13.1015	6.49382	-1.01259	0.0104658
CSNK1E	28.8484	14.2994	-1.01254	0.00414075
DMWD	17.2583	8.55613	-1.01226	0.00166463
RFNG	36.6296	18.1627	-1.01203	0.00618756
KLC2	11.1496	5.5307	-1.01145	0.00709292
PLXNA2	18.17	9.01498	-1.01116	0.00166463
GTF2F1,MLLT1	44.8096	22.2607	-1.00931	0.0181454
CLIP2	3.34169	1.66028	-1.00915	0.0052039
PDXK	44.0479	21.885	-1.00913	0.00618756
ATG4B	8.51035	4.22842	-1.0091	0.0439079
SCN2B	1.5182	0.754906	-1.00799	0.0277976
NECAP2	10.2684	5.11184	-1.0063	0.0319719
TIAM2	7.21301	3.59117	-1.00615	0.00618756
ZHX2	4.60896	2.29534	-1.00573	0.0175929
CIB2	36.3063	18.084	-1.00551	0.0052039
PALM2AKAP2	83.0305	41.405	-1.00384	0.0222118
ZMIZ1	18.1059	9.03006	-1.00366	0.00166463
ZNF618	1.09783	0.547785	-1.00297	0.0228621
STC1	60.2396	30.0602	-1.00286	0.00970753

KATNB1	5.81698	2.90344	-1.00251	0.0142522
RASL11B	8.68378	4.33553	-1.00211	0.0156032
WWC3	27.4498	13.7141	-1.00113	0.00166463
SPP1	50.6374	25.3106	-1.00046	0.00166463
CCM2	10.3313	5.16558	-1.00002	0.011901
UBASH3B	1.83401	0.917018	-0.999982	0.0324006
ENSCAFG00000049822	12.6845	6.34672	-0.998983	0.00970753
RASIP1	12.9024	6.45677	-0.998751	0.00709292
MBNL2	86.0167	43.0487	-0.998646	0.0104658
INHA	5.55582	2.781	-0.998395	0.0388821
ARMC5	1.24767	0.624555	-0.998333	0.0348071
CD81	367.993	184.211	-0.998322	0.00166463
CELSR1	8.2897	4.14992	-0.998236	0.0104658
IDO1	16.454	8.23805	-0.998067	0.0198195
PTPRU	1.20283	0.602866	-0.996527	0.0204062
KIAA1755	1.61928	0.811868	-0.996034	0.0351794
ENSCAFG00000023602	29.2844	14.6846	-0.995826	0.0338795
ENSCAFG00000043507	15.4604	7.76529	-0.993465	0.0348071
LRRC32	3.77564	1.89671	-0.993221	0.0104658
ENSCAFG00000049186	57.7966	29.0388	-0.993002	0.0104658
RELB	17.0311	8.55887	-0.992683	0.00709292
ACTG1	231.077	116.13	-0.992632	0.00166463
RGS3	13.9294	7.00429	-0.99182	0.0111445
APOL6	8.89742	4.47776	-0.990608	0.0111445
CKAP4	23.5196	11.8421	-0.989939	0.00166463
FSTL1	114.166	57.4923	-0.989697	0.00166463
PCBP4	24.8466	12.5143	-0.989472	0.00298108
SMTNL2	5.58093	2.81138	-0.989228	0.0142522
ABI3	3.95378	1.99443	-0.987254	0.0338795
MAP1A	20.5905	10.3928	-0.986387	0.00166463
PPM1F	6.02684	3.04589	-0.98454	0.00166463
PLEKHO2	10.6308	5.37277	-0.98451	0.00618756
ENSCAFG00000018211	2.35173	1.18885	-0.984156	0.0288776
FKBP1A	23.6665	11.9735	-0.982996	0.00799923
PI4KA	18.8088	9.51674	-0.982865	0.00618756
SMPD4	7.366	3.72723	-0.982779	0.0052039
NPRL3	4.05267	2.05132	-0.982319	0.0313973
JDP2	10.6319	5.38516	-0.981339	0.0324006
GRN	21.2219	10.7543	-0.98064	0.00414075
CTPS1	5.92209	3.00109	-0.980618	0.0135044
DHX37	5.50531	2.78992	-0.980599	0.0052039
PRKCSH	20.6673	10.4746	-0.980456	0.00414075
SMPDL3A	25.9639	13.1629	-0.980032	0.0175929
VWF	127.135	64.487	-0.979281	0.00166463
SHROOM2	7.61545	3.863	-0.979206	0.00166463
PIM1	5.23511	2.65588	-0.979031	0.00709292
KIAA0753	2.84115	1.44253	-0.977871	0.011901
ASPSCR1	54.9734	27.9351	-0.976656	0.00166463
LCP1	8.8336	4.48893	-0.97663	0.0052039
FAM89B	17.1177	8.70307	-0.975891	0.0150001
ARHGDIB	33.1624	16.8623	-0.97575	0.0142522
SCLY	9.74191	4.96493	-0.972431	0.0175929
ADAMTS1	66.04	33.6814	-0.971388	0.00166463

LAMTOR2	75.9866	38.7663	-0.97094	0.00618756
ARNT,CTSK	93.7015	47.8286	-0.9702	0.0052039
FURIN	23.7253	12.1107	-0.970141	0.00298108
VGLL4	10.7037	5.46431	-0.969996	0.0245705
MAST2	33.6358	17.1836	-0.968966	0.00166463
CHMP1A	17.3097	8.84644	-0.96841	0.0104658
QTRT1	7.9089	4.04441	-0.967548	0.0411794
LGALS1	198.298	101.447	-0.966944	0.00166463
RNH1	14.5035	7.42209	-0.966501	0.0435493
P2RY6	24.6654	12.6338	-0.965203	0.0384185
MGLL	48.3647	24.7877	-0.964329	0.0104658
CLMN	3.70335	1.89924	-0.963409	0.02735
ENSCAFG00000003386	193.583	99.2821	-0.963347	0.00166463
RBM38	32.0115	16.4244	-0.962755	0.0192697
ADCY4	33.5066	17.2096	-0.961229	0.00166463
PRDM1	1.36912	0.70327	-0.961093	0.0366339
TAPBP	22.7036	11.6655	-0.960676	0.00166463
UCP2	15.8779	8.16113	-0.960176	0.00970753
ABTB2	3.39332	1.746	-0.958642	0.0257323
INCENP	2.2519	1.15871	-0.958624	0.0388821
HSPB8	927.357	477.228	-0.958448	0.00166463
ITM2C	10.7833	5.55006	-0.958218	0.00414075
EMP3	20.6786	10.6507	-0.957195	0.0257323
POR	22.8947	11.7932	-0.957064	0.00414075
ACVRL1	16.3335	8.4231	-0.955413	0.0181454
PEF1	42.7924	22.0808	-0.954565	0.0388821
OAS2	14.9336	7.70658	-0.954401	0.0198195
PTOV1	38.9009	20.0927	-0.953135	0.00166463
FARSA	12.4892	6.45203	-0.952859	0.0104658
STK40	23.1417	11.964	-0.951802	0.0198195
GALNT18	2.46712	1.27553	-0.951731	0.0370398
ENSCAFG00000029293,ENSCAFG00000043957	6.60072	3.41307	-0.951554	0.0293656
ADGRB1	2.67641	1.38421	-0.951233	0.0263476
TP53	24.8927	12.8929	-0.949148	0.00298108
DDAH2	9.56969	4.96041	-0.948012	0.0240207
BIRC3	114.279	59.2464	-0.947756	0.0181454
DPH1,OVCA2	9.64548	5.00252	-0.947199	0.0370398
PIN1	30.1653	15.6622	-0.945603	0.0156032
NOP2	9.36373	4.86274	-0.945313	0.011901
C1S	22.6011	11.7407	-0.944868	0.00298108
MAP3K14	3.71672	1.93123	-0.94451	0.0186537
CLTA	125.352	65.1746	-0.943597	0.00414075
ATP13A1	6.2991	3.27515	-0.943585	0.00709292
CNPY3	8.70551	4.53012	-0.942379	0.0304032
ABCA3	1.44599	0.752731	-0.941853	0.044199
GSTP1	102.854	53.5448	-0.941782	0.00298108
KLHL36	3.61743	1.88368	-0.941414	0.0324006
WARS1	113.622	59.184	-0.94096	0.00166463
FZR1	12.8908	6.71767	-0.940307	0.0298794
WFS1	23.2618	12.1324	-0.939093	0.00298108
B4GALT7	8.94142	4.66846	-0.937559	0.0277976
TRIP6	9.43502	4.92833	-0.936926	0.0142522
CDR2L	5.43937	2.84217	-0.936448	0.00970753

TFPI2	21.5553	11.2632	-0.93643	0.0450444
OSMR	33.6338	17.5858	-0.9355	0.00414075
RABAC1	90.4125	47.3084	-0.934426	0.00298108
SLC4A2	7.4802	3.9181	-0.932925	0.0487642
BCL2L11	5.96277	3.12505	-0.932102	0.02735
RRP9	8.35181	4.37886	-0.931535	0.030955
SLC2A5	25.4593	13.3513	-0.931213	0.02735
EFS	1.01552	0.532595	-0.931112	0.0454727
AXIN1	6.54301	3.43188	-0.930958	0.0175929
ENSCAFG00000003026	44.2202	23.2058	-0.930218	0.0150001
ZFP36L1	40.0751	21.0477	-0.929046	0.00414075
PLEKHG5	4.6737	2.455	-0.928841	0.0135044
KIF1C	297.293	156.163	-0.928831	0.00709292
ARAF	55.2198	29.0566	-0.926318	0.0277976
ADAP2	2.80122	1.47499	-0.925348	0.0186537
CALM3	128.406	67.6868	-0.923767	0.0222118
LZTS2	11.1068	5.85483	-0.923737	0.0156032
CSF1	46.2813	24.4345	-0.921509	0.0170082
SPECC1	2.88544	1.52441	-0.920545	0.0348071
SH2D3C	14.77	7.80397	-0.920386	0.00414075
NES	65.2554	34.523	-0.918539	0.00166463
TGFB1	16.0169	8.47875	-0.917672	0.0192697
GGA1	13.66	7.2382	-0.916254	0.00799923
SLC35D1	35.1656	18.646	-0.915294	0.00166463
IMPDH1	16.3452	8.67244	-0.914361	0.0192697
NEDD9	62.1062	32.9541	-0.914278	0.00166463
SDC4	99.5207	52.8391	-0.91339	0.0175929
SORL1	0.824216	0.437615	-0.913361	0.0379891
GRB2	46.0487	24.4774	-0.911706	0.0304032
CCDC86	21.7612	11.5746	-0.910794	0.0370398
FOXP4	4.83813	2.57468	-0.910058	0.0460276
EHD2	31.0783	16.5424	-0.90974	0.00166463
LRRC59	12.7943	6.81177	-0.9094	0.0052039
ASB15,LMOD2	1607.99	857.656	-0.906786	0.0411794
AFAP1L1	37.3118	19.9295	-0.904729	0.008824
WBP1	27.0085	14.4263	-0.90471	0.0135044
PCDH18	3.43081	1.83312	-0.90425	0.0228621
RRP1B	7.59934	4.0617	-0.90379	0.0216077
RAB11FIP3	18.0907	9.66924	-0.903774	0.00799923
TAZ	30.5058	16.307	-0.90359	0.0052039
CSNK1G1	59.0956	31.6003	-0.903111	0.0192697
SLIT2	1.73193	0.926459	-0.902583	0.026814
INPPL1	51.2628	27.4403	-0.901617	0.00166463
NT5DC3	20.3717	10.9068	-0.901344	0.00298108
ELMSAN1	8.03607	4.30308	-0.901122	0.00166463
CLU	54.5013	29.1977	-0.900436	0.00166463
MRPL28	34.6941	18.6169	-0.898079	0.00709292
CUEDC1	22.8244	12.2479	-0.898046	0.0186537
DRAP1	39.186	21.0287	-0.897982	0.0052039
ACTR1B	58.8149	31.5712	-0.897573	0.00166463
EZR	104.974	56.3492	-0.897568	0.00166463
P4HA2	15.1185	8.1194	-0.89687	0.0150001
FITM1	31.1818	16.7549	-0.896119	0.0186537

ENSCAFG00000044698	33.6038	18.0564	-0.896117	0.0313973
ZDHHC18	14.7713	7.94093	-0.895422	0.0135044
ITIH4	5.74254	3.08716	-0.895408	0.0235189
HSPA12B	13.2247	7.11469	-0.894362	0.0333266
RPL28	299.401	161.082	-0.894284	0.0052039
ANKRD39	583.102	313.825	-0.893786	0.026814
MVP	22.6716	12.2066	-0.893223	0.0052039
ARF1	81.1442	43.6984	-0.892909	0.0288776
CYSTM1	132.462	71.3655	-0.89228	0.0127236
IL17C,ZC3H18	8.07547	4.35377	-0.891283	0.0186537
YIF1A	26.3771	14.2328	-0.89006	0.0150001
ZNF316	16.5426	8.92925	-0.889573	0.0375549
SFRP1	11.6068	6.26788	-0.88892	0.0425903
EPB41L2	11.6592	6.30113	-0.887785	0.00709292
KLF4	17.5306	9.47699	-0.887378	0.0156032
UBXN6	35.4333	19.1578	-0.887173	0.00618756
FBL	88.239	47.7152	-0.886967	0.0379891
INTS1	7.04131	3.80781	-0.886883	0.00799923
DPT	150.923	81.6368	-0.886519	0.00166463
DGUOK	641.34	346.933	-0.886429	0.00166463
GABBR1	11.9127	6.445	-0.886247	0.0127236
MICAL3	16.2704	8.80683	-0.885555	0.00298108
C28H10orf71	123.872	67.072	-0.885063	0.00298108
DGKI	20.483	11.1034	-0.883427	0.00414075
MYL9	272.356	147.662	-0.8832	0.00166463
MTHFD1L	3.75275	2.03506	-0.882875	0.0361288
MANF	62.5488	33.9763	-0.880455	0.00618756
MCRS1	15.0518	8.17776	-0.880162	0.0186537
SF3B4	8.85333	4.8144	-0.878863	0.0324006
TJAP1	5.19634	2.82899	-0.877206	0.0293656
SEPTIN9	62.1656	33.9352	-0.873333	0.00298108
ENSCAFG00000046342	41.1326	22.4653	-0.872585	0.0181454
STK25	29.4805	16.1098	-0.871819	0.00414075
TLE2	24.0928	13.1667	-0.871703	0.00799923
MSX1	8.59323	4.69627	-0.871684	0.0439079
SPSB1	8.31952	4.54808	-0.871242	0.0175929
CLIC1	35.9802	19.6763	-0.870744	0.0111445
CFL1	83.2734	45.55	-0.870405	0.0150001
ELL	76.401	41.7919	-0.870369	0.0288776
SPRYD3	9.25671	5.06934	-0.868702	0.0366339
ENSCAFG00000004446	24.3333	13.3376	-0.867436	0.00618756
USF1	16.4213	9.00145	-0.867339	0.0150001
HPCAL1	11.1744	6.12815	-0.86667	0.0163086
PSD4	5.21468	2.8617	-0.865708	0.0156032
ENSCAFG00000044521	4.50827	2.47417	-0.86563	0.0293656
ANGPT4	2.2326	1.22569	-0.86513	0.0496526
SAMD4A	96.1777	52.8234	-0.864525	0.0263476
MAFB	4.74203	2.60743	-0.862877	0.026814
LDB3	772.871	425.12	-0.862359	0.00970753
UNC45B	70.8338	38.9892	-0.861364	0.00414075
SLC38A2	89.094	49.0508	-0.861054	0.0204062
CNKSR3	17.8818	9.84569	-0.860931	0.0127236
S1PR1	44.1906	24.3328	-0.860839	0.00709292

DLA-DQA1	16.6058	9.14421	-0.860756	0.0111445
NFKBIB	10.0794	5.55406	-0.859803	0.0464503
BSG	165.261	91.1361	-0.858653	0.0052039
CA4	27.4703	15.1659	-0.857037	0.0251707
MAN1C1	6.34655	3.50478	-0.85665	0.0379891
KLHL3	16.7548	9.25664	-0.856012	0.00970753
TSPAN9	36.8465	20.3653	-0.855418	0.00166463
CCND1	15.2158	8.41541	-0.854469	0.00414075
ENSCAFG00000003880	19.0462	10.534	-0.854454	0.00970753
RBCK1	14.4061	7.97126	-0.853797	0.0175929
ARID5B	28.4243	15.7324	-0.853391	0.00298108
XPC	10.9091	6.04291	-0.852224	0.0150001
TMEM11	37.9895	21.0649	-0.850762	0.0283656
AARS2	4.92696	2.73251	-0.850471	0.0240207
PLIN5	27.1031	15.0323	-0.850388	0.011901
CAVIN1	95.1672	52.8013	-0.84989	0.00166463
ENSCAFG00000044070	31.3594	17.4012	-0.849712	0.044199
PTP4A3	1115.84	619.584	-0.848752	0.0175929
ENSCAFG00000014422	19.0662	10.5879	-0.848605	0.0052039
CTNND1	31.983	17.7615	-0.848553	0.00709292
CCDC97	6.16167	3.42427	-0.847523	0.026814
ACTA2	57.2194	31.8362	-0.845835	0.0052039
IKBKB	15.0772	8.38928	-0.845748	0.0127236
NID1	40.8881	22.7518	-0.8457	0.00298108
EPB41L1	8.34424	4.6444	-0.845288	0.0135044
UNC45A	7.29764	4.06248	-0.845069	0.0127236
BDH1	16.6118	9.25044	-0.844614	0.0175929
CYREN,WDR91	44.6776	24.88	-0.844563	0.0251707
CCDC80	25.4974	14.2029	-0.84417	0.00618756
MAP3K3	27.9552	15.5723	-0.844136	0.00799923
TWF2	36.9801	20.6048	-0.843767	0.0175929
SELENOW	292.681	163.245	-0.842289	0.00166463
STKLD1	10.4804	5.84615	-0.842137	0.026814
UNC5A	9.83863	5.48838	-0.842076	0.0175929
WNK2	11.7195	6.54059	-0.841417	0.0170082
ITGA5	91.8708	51.3092	-0.84039	0.008824
CARD6	13.0994	7.31656	-0.84026	0.0104658
SP2	7.67339	4.29031	-0.838785	0.0304032
NCOR2	11.947	6.68576	-0.837486	0.00166463
JAG1	11.2748	6.31206	-0.836913	0.0142522
OBSL1	73.1727	40.9742	-0.836588	0.0209928
PPP1R14B	97.9582	54.8533	-0.836588	0.0186537
MX2	22.8054	12.7843	-0.835008	0.0142522
RAPSN	11.7829	6.60787	-0.834441	0.0361288
SEMA7A,UBL7	80.76	45.2913	-0.834407	0.00618756
CLCN4	30.4756	17.0978	-0.833841	0.0216077
ZCCHC24	27.6152	15.5021	-0.832999	0.0150001
JUN	214.576	120.455	-0.832993	0.0283656
GPT	55.2162	31.0124	-0.832245	0.0175929
ALPK2	68.2758	38.3994	-0.830291	0.00166463
CMIP	10.2159	5.74946	-0.829323	0.0104658
TRAFD1	54.7051	30.792	-0.82912	0.00618756
ATG2A	3.12317	1.75816	-0.828948	0.0324006

PINK1	121.861	68.6145	-0.828651	0.0127236
CTBP1	20.7505	11.6842	-0.828593	0.0156032
ENSCAFG00000042598	664.48	374.741	-0.826333	0.0135044
SMAD3	14.87	8.38997	-0.825668	0.0052039
NUDT16L1	17.1986	9.70601	-0.825338	0.044199
TMEM132A	41.7512	23.5626	-0.825321	0.00799923
MYOF	2.88267	1.62753	-0.82472	0.0361288
AP2A1	27.1448	15.3515	-0.822299	0.00709292
MYH14	13.1558	7.44025	-0.822279	0.0487642
KMT2D	6.41031	3.62611	-0.821972	0.008824
SLC25A25	27.5405	15.5797	-0.82189	0.0170082
ENO1	148.913	84.2525	-0.821676	0.00414075
PCSK6	6.92324	3.91707	-0.821671	0.0186537
ENSCAFG00000016640,TMEM265	13.8007	7.80903	-0.821528	0.00166463
WDR1	60.0154	33.9705	-0.821049	0.00298108
SEMA4D	8.52812	4.83011	-0.820173	0.0181454
TINAGL1	98.5995	55.8971	-0.818806	0.0135044
ETS2	27.6316	15.6674	-0.81855	0.0324006
SF3A2	10.3518	5.86996	-0.818454	0.0277976
DPP9	10.7597	6.10273	-0.818113	0.026814
PDK4	1057.24	599.697	-0.817993	0.0348071
FAM114A1	6.37243	3.61542	-0.817679	0.0450444
KIT	5.11631	2.90309	-0.817518	0.0411794
P3H1	9.09977	5.16566	-0.816878	0.0351794
PPP2R1A	103.465	58.7585	-0.816272	0.00799923
SIPA1L1	3.23358	1.83652	-0.81616	0.0333266
MYO1E	22.9617	13.0443	-0.815808	0.0142522
B4GALT5	46.1075	26.2012	-0.815369	0.0204062
SGTA	28.6403	16.2761	-0.815294	0.0425903
ENSCAFG00000019869	8.31172	4.7267	-0.814312	0.0198195
SHC1	15.8196	9.00037	-0.813657	0.0251707
PARVB	49.6567	28.2594	-0.813258	0.0186537
SZT2	2.78399	1.58539	-0.812317	0.0277976
PTGER4	27.8644	15.8706	-0.812066	0.00799923
TONSL,VPS28	53.0229	30.224	-0.810919	0.0240207
APLP2	212.847	121.362	-0.810505	0.00618756
ARHGEF10L	7.61615	4.34331	-0.810266	0.0319719
NPTN	120.766	68.8898	-0.809848	0.0156032
TMEM184B	27.0108	15.4234	-0.808419	0.044634
ANKH	35.8382	20.4644	-0.808386	0.0163086
COL14A1	18.6498	10.6494	-0.808386	0.0370398
KIAA2013	11.4773	6.55795	-0.807473	0.0328579
C3	10.6848	6.10644	-0.807158	0.0192697
RAB3IL1	122.485	70.035	-0.806458	0.00298108
LENG8	34.9767	20.0321	-0.804081	0.00799923
FAM20C	8.93622	5.11809	-0.804059	0.0204062
SARDH	7.83338	4.49058	-0.802734	0.0343735
C14H1orf35	28.1855	16.1665	-0.801949	0.0235189
STX6	24.6016	14.123	-0.800706	0.0304032
PRKAG3	147.725	84.8454	-0.8	0.008824
TIE1	7.69966	4.4226	-0.799901	0.0288776
PDGFRB	21.9649	12.6191	-0.799589	0.0240207
HEG1	29.5731	16.9933	-0.799316	0.0170082

RRAD	139.146	79.9887	-0.798736	0.011901
MYOM3	168.655	96.9688	-0.798479	0.00709292
SLC2A1	20.9329	12.037	-0.798299	0.00799923
CD163	12.8448	7.39381	-0.796789	0.0192697
ECE1	51.7297	29.7795	-0.796673	0.00799923
ETV6	16.3107	9.39314	-0.796139	0.0298794
PPP1R11	36.6465	21.1066	-0.795983	0.0240207
NGFR	3.264	1.881	-0.795143	0.0356752
GOLM1	17.9194	10.3269	-0.795114	0.0240207
SIAH2	15.7008	9.05137	-0.794634	0.0209928
SNTA1	150.026	86.5443	-0.793706	0.00618756
PKN1	14.0751	8.12727	-0.792302	0.0398565
RAPGEF5	10.6328	6.14243	-0.791642	0.0104658
PTPN1	24.47	14.1398	-0.791259	0.0235189
BCKDK	57.87	33.4509	-0.790771	0.00970753
JUP	29.7837	17.2194	-0.790488	0.00970753
SLC12A4	26.2965	15.2116	-0.789695	0.008824
VASH2	10.3647	6.00061	-0.788501	0.0216077
BCL6B	6.58008	3.81572	-0.786151	0.0351794
RING1	17.6544	10.2384	-0.786043	0.0277976
MSN	102.742	59.5947	-0.785765	0.0127236
GYS1	39.4996	22.9336	-0.784376	0.00618756
CD34	66.098	38.3773	-0.784353	0.0052039
DAXX	6.60421	3.83755	-0.7832	0.044199
CALR	239.226	139.055	-0.782722	0.00166463
POLR2A	20.8925	12.1599	-0.78085	0.0052039
DNAJB12	14.5264	8.46202	-0.779599	0.0475889
LAMA2	141.2	82.3275	-0.77829	0.0142522
ATXN1L	12.8351	7.49233	-0.776612	0.00618756
FRMD4A	15.8843	9.2841	-0.774768	0.0127236
ENSCAFG00000007026	64.8418	37.9685	-0.772122	0.0216077
CHD7	10.8054	6.3295	-0.77159	0.0111445
MYO9B	3.72183	2.18114	-0.77093	0.0293656
UPF1	11.1605	6.54445	-0.770059	0.0192697
MAPK8IP3	7.82948	4.60507	-0.765695	0.0388821
TRIR	60.1139	35.3646	-0.765394	0.0222118
MYO18B	121.757	71.6375	-0.765219	0.00166463
ECI1	58.8834	34.6816	-0.763691	0.0304032
CD79A	481.07	283.701	-0.761876	0.008824
TPM4	165.239	97.4505	-0.76181	0.0204062
HSPA2	23.5774	13.9095	-0.761336	0.0228621
ATP8B1	9.78208	5.7759	-0.760095	0.02735
TMEM143	19.677	11.6195	-0.759966	0.0351794
AP1M1	18.2059	10.7597	-0.758766	0.0450444
RALY	62.1928	36.8324	-0.755771	0.0209928
VIM	229.953	136.618	-0.751189	0.0052039
ENSCAFG00000013413	95.8369	56.941	-0.751115	0.0156032
CORO6	42.6022	25.3142	-0.750979	0.0216077
ARHGAP17	13.2535	7.87543	-0.750941	0.0348071
ENSCAFG00000001854	104.935	62.3554	-0.750914	0.0181454
ARHGEF17	12.577	7.47529	-0.750588	0.0209928
TAP1	22.931	13.6359	-0.749891	0.0408367
PLCXD3	27.387	16.2925	-0.749285	0.0156032

PRSS23	7.67367	4.5652	-0.749237	0.0431027
TLN1	50.2921	29.9376	-0.748373	0.0104658
MAPK3	15.1388	9.01946	-0.747134	0.0487642
CYTH1	45.6762	27.2233	-0.746601	0.0351794
SERPING1	37.8825	22.5839	-0.746234	0.0222118
ENSCAFG00000006693	35.077	20.9147	-0.746008	0.0333266
CALD1	45.8466	27.3414	-0.745731	0.0421371
AQP1	126.97	75.7361	-0.745435	0.0111445
LETM1	21.4321	12.8032	-0.743263	0.0313973
MYO10	18.9884	11.3575	-0.741476	0.0170082
KERA	26.2647	15.7284	-0.739758	0.0333266
RPL3	60.1483	36.0349	-0.73913	0.0181454
LONP1	27.5086	16.4805	-0.739123	0.0484275
PLIN2	93.359	55.9792	-0.7379	0.0198195
TBC1D1	17.1458	10.2809	-0.737887	0.0163086
HIP1	36.5588	21.9453	-0.736305	0.00970753
RSPH6A,SYMPK	16.557	9.95989	-0.733244	0.0198195
PSMD8	111.994	67.3897	-0.732815	0.0283656
CIAO2B	93.6717	56.3756	-0.732543	0.039381
PHRF1	5.69039	3.42688	-0.731633	0.0408367
PELP1	11.6952	7.05339	-0.72953	0.0328579
CAPN2	35.609	21.4806	-0.729208	0.0313973
TRIM54	197.193	119.085	-0.727611	0.0186537
CARS1	11.234	6.78904	-0.726596	0.0491872
RPL3L	225.352	136.292	-0.725481	0.0384185
BAG6	47.6675	28.8632	-0.723773	0.0170082
CDC34	132.838	80.5282	-0.722098	0.02735
SSH1	13.2615	8.03995	-0.721982	0.0156032
PTPN21	40.0727	24.3194	-0.720512	0.0484275
SIDT2	17.0715	10.3639	-0.720026	0.0361288
CSTB	124.956	75.8869	-0.7195	0.044634
TOP3B	33.9636	20.6311	-0.71917	0.0245705
SMARCA4	10.7744	6.5625	-0.715289	0.0277976
PUF60	66.5843	40.6372	-0.71238	0.0170082
MRAS	36.1617	22.0726	-0.712208	0.0404086
SYNPO2L	406.703	248.317	-0.71179	0.0181454
NUCB1	60.5341	36.9648	-0.711598	0.0235189
ZMYND8	6.88263	4.20949	-0.709314	0.0379891
AGPAT2	79.682	48.747	-0.70894	0.0263476
KHSRP	16.0967	9.85093	-0.708433	0.0484275
MTOR	27.9345	17.0979	-0.70823	0.02735
DHX30	11.752	7.20249	-0.706335	0.0471762
SPTB	40.3066	24.7193	-0.70538	0.011901
DDX23	14.376	8.821	-0.704648	0.0324006
RHOJ	54.5883	33.5449	-0.702499	0.0384185
GNG7	23.7523	14.6245	-0.699684	0.0411794
ZC3H7B	18.9247	11.6556	-0.699242	0.0450444
ARPC1A	39.1216	24.1347	-0.696856	0.0343735
PLXND1	8.59997	5.30603	-0.696698	0.0351794
UBA1	58.014	35.7983	-0.696508	0.0156032
FLII	47.7059	29.4968	-0.693607	0.0111445
TNRC6C	8.60342	5.32549	-0.691995	0.0435493
CREBBP	8.45504	5.23568	-0.691435	0.0361288

EIF4G1	81.7909	50.8037	-0.687006	0.0163086
HADH	457.387	284.196	-0.686529	0.0263476
NCSTN	45.7929	28.4633	-0.686021	0.0251707
MOV10	29.0267	18.0451	-0.685772	0.0319719
NDRG1	37.1898	23.1513	-0.683811	0.039381
LAMC1	131.301	81.8307	-0.682165	0.0181454
ACADS	72.0156	44.8854	-0.682064	0.0356752
BRD2	70.0045	43.6451	-0.681627	0.0198195
KDM5C	15.3556	9.5879	-0.679474	0.0475889
NDST1,SYNPO	116.192	72.7215	-0.676053	0.0240207
PALLD	163.742	102.79	-0.671727	0.0487642
ASXL1	12.4576	7.83451	-0.669107	0.0475889
ENSCAFG00000015122	204.275	128.494	-0.668812	0.0240207
LRRFIP1	118.731	74.837	-0.665878	0.0324006
NFIL3	51.6006	32.66	-0.659865	0.0388821
TUBA1A	85.8875	54.5971	-0.653623	0.0421371
PRPF19	27.1415	17.2742	-0.651877	0.0468698
ZNFX1	19.8565	12.66	-0.64934	0.030955
ENSCAFG00000019472	483.792	308.489	-0.649166	0.0304032
ENSCAFG0000006102	231.699	147.831	-0.6483	0.0293656
SLC39A14	69.3052	44.2566	-0.64707	0.0240207
ITGB5	68.5787	43.8122	-0.646426	0.0480651
TGFBR2	18.0208	11.518	-0.645771	0.0431027
LAMA4	81.6998	52.2592	-0.644647	0.0245705
ATP2B4	27.9234	17.8841	-0.642798	0.0257323
ENSCAFG00000029163	882.294	565.271	-0.642318	0.0313973
NOP53	29.7151	19.0463	-0.641689	0.044199
ADAR	15.9617	10.2498	-0.639019	0.0496526
GPRC5B	64.7945	41.7109	-0.635447	0.026814
CDH5	35.7329	23.1258	-0.627749	0.0435493
SKI	16.7749	10.8669	-0.626363	0.0416803
ITGA9	15.2054	9.85148	-0.62617	0.0348071
TANC1	36.6334	23.7723	-0.62388	0.0388821
SLC25A6	123.77	80.9015	-0.613423	0.0475889
CD63	265.087	173.762	-0.609349	0.0435493
ARID1A,PIGV	13.4754	8.86929	-0.603437	0.0471762
RPS7	435.501	289.044	-0.59139	0.0384185
ZC3H15	54.4715	83.3283	0.613304	0.0439079
ALDH6A1	115.684	178.853	0.628589	0.0283656
MKKS	28.3496	43.9606	0.632882	0.0468698
ATP5PB	1147.64	1781.61	0.634508	0.0468698
PERP	120.668	187.801	0.638159	0.0235189
MCFD2	41.169	64.3238	0.643794	0.0313973
AKAP9	9.40453	14.9784	0.67146	0.0240207
ENSCAFG00000017667	293.164	470.039	0.681075	0.011901
ADH4	21.7775	35.1011	0.688682	0.0450444
FBN2	8.38721	13.5303	0.689935	0.0324006
BTBD8,C6H1orf146	21.2894	34.3562	0.690431	0.0338795
ENSCAFG00000042602	37.2941	60.3684	0.694845	0.0411794
MYLK3	131.853	213.634	0.69621	0.0127236
GADD45A	116.037	188.239	0.697983	0.0175929
ADD3	33.8739	55.1789	0.703944	0.0245705
RB1CC1	14.0068	22.8642	0.706964	0.0228621

PRKDC	1.92164	3.14192	0.709306	0.0408367
DGKH	12.036	19.7151	0.711952	0.0170082
MDH1	1153.77	1892.11	0.713636	0.0361288
MTX2	52.7816	86.9132	0.719539	0.02735
PSMG2	45.6307	75.1616	0.71999	0.0245705
GHR	49.5443	82.3446	0.732956	0.0257323
ENSCAFG00000047391	32.7217	54.3906	0.733109	0.0175929
PDP1	16.0849	26.7461	0.733623	0.0460276
DARS1	26.4654	44.023	0.734149	0.0156032
CRIPT	22.8746	38.2488	0.74167	0.0471762
ENSCAFG00000000713	43.898	73.4643	0.742888	0.0150001
OSBPL1A	5.71706	9.57063	0.743342	0.0283656
NAA16	15.3962	25.8063	0.745156	0.030955
GCSH	112.567	189.25	0.749511	0.0216077
EPM2AIP1	10.5732	17.8012	0.751563	0.00709292
TSHB	29.4502	49.6272	0.752854	0.0496526
LIFR	4.66339	7.8586	0.752892	0.00970753
FGF1	22.4957	37.9197	0.753299	0.0370398
ZYG11B	20.4179	34.4906	0.756368	0.0240207
ENSCAFG00000003760	45.4	76.708	0.756685	0.0156032
TECRL	321.008	547.014	0.768967	0.0181454
CMYA5	142.308	242.645	0.769828	0.0181454
RBM34	30.6125	52.1994	0.769911	0.00709292
ENSCAFG00000030982	15.1466	25.8945	0.773654	0.0156032
PERM1	30.6574	52.4602	0.774988	0.0111445
CCNDBP1,TMEM62	29.9871	51.3559	0.776188	0.0192697
KYAT1	23.5142	40.2903	0.7769	0.0111445
PDE1C	95.3943	163.863	0.780518	0.0150001
KCNE2	8.54371	14.7355	0.786361	0.0111445
ZNF140,ZNF84	10.143	17.499	0.786783	0.0439079
MTRF1	11.9175	20.5915	0.788964	0.044634
RIC8B	5.94022	10.299	0.793915	0.044199
RHPN2	4.14882	7.1951	0.794314	0.0333266
P2RY1	14.9661	25.9685	0.795066	0.0298794
TIGAR	14.8986	25.901	0.797826	0.0135044
BHLHB9	17.009	29.5747	0.798064	0.0257323
ENSCAFG00000001397	24.2316	42.1427	0.798394	0.0324006
TBX20	57.1599	99.5018	0.799721	0.00618756
CDC26	15.6962	27.3602	0.801664	0.044634
AKAP11	4.3499	7.63177	0.811037	0.00970753
ENSCAFG00000016072	34.6423	60.8768	0.813357	0.00709292
ADGRV1	7.17486	12.6176	0.814418	0.0366339
COX3,MT-ATP6,MT-ATP8	1782.43	3148.16	0.820666	0.00709292
RMDN2	6.41033	11.3736	0.827219	0.0431027
ADAM32	2.81298	4.99671	0.828877	0.0439079
HPF1	8.10609	14.4014	0.829128	0.0328579
SLC1A2	3.05582	5.44435	0.833198	0.0204062
ENSCAFG00000044475	1020.75	1824.92	0.838208	0.0052039
ENSCAFG00000042877	1295.45	2317.73	0.839261	0.00166463
NUAK2	5.83156	10.4759	0.845127	0.0324006
ENSCAFG00000042998	32.6593	58.7735	0.847673	0.0356752
IVNS1ABP	553.26	995.89	0.848028	0.011901
FBXL5	17.4233	31.5098	0.854783	0.0277976

TMEM126A	47.5408	86.2742	0.859763	0.0111445
TIMM8A	16.2115	29.508	0.86409	0.0104658
ZNF667	2.40627	4.38102	0.864467	0.044634
ENSCAFG00000028987,ENSCAFG00000029302	92.6631	168.759	0.8649	0.0198195
PTPN3	57.793	105.747	0.871647	0.00970753
STEAP2	2.56258	4.70368	0.87619	0.0104658
ENSCAFG00000005585	58.657	107.666	0.876192	0.00414075
MLIP	112.598	207.142	0.87944	0.008824
NR4A3	9.80712	18.0824	0.882689	0.0052039
TMEM67	0.953366	1.75809	0.882907	0.0491872
ASB10	6.25711	11.5854	0.888743	0.0163086
ENSCAFG00000007165	6.12036	11.3865	0.895642	0.0283656
ERBB4	0.72592	1.352	0.897216	0.0435493
BPHL	9.87655	18.3973	0.897417	0.0186537
RAB9B	1.09884	2.04794	0.898198	0.0421371
CLDN10	15.0242	28.0112	0.898716	0.0204062
FPGT	2.21624	4.13621	0.900194	0.0348071
FAM214A	17.2515	32.3036	0.904973	0.00166463
TRPC1	7.35426	13.795	0.907496	0.0111445
FAM81A	2.34716	4.40733	0.908988	0.0435493
MTERF1	2.72535	5.13008	0.912539	0.0288776
NEIL2	3.169	5.96957	0.913599	0.0375549
SHISAL1	2.89467	5.45281	0.913601	0.039381
SLF2	3.11	5.85846	0.913606	0.0142522
CCDC28A	10.8326	20.4587	0.917336	0.0135044
PROX1	12.5472	23.7217	0.918845	0.0111445
GPD2	6.31661	11.948	0.91955	0.00166463
ZFP28	2.33736	4.42662	0.921325	0.0163086
ZFP30	1.89489	3.59914	0.92554	0.0454727
CCNG2	9.78132	18.6041	0.927523	0.0343735
ASB18	21.7937	41.6017	0.932731	0.00166463
NPY1R	2.14856	4.10468	0.933898	0.044634
ENSCAFG00000043540	11.3384	21.6623	0.933967	0.00618756
ZNF583	1.53759	2.9562	0.943071	0.0384185
BDH2	5.99119	11.5746	0.950055	0.030955
RMI1	3.50471	6.79017	0.954155	0.0216077
ENSCAFG00000042572	344.794	668.931	0.956125	0.00166463
PREP	16.5068	32.0345	0.956564	0.0411794
MAMSTR	2.21403	4.30408	0.959026	0.0163086
PFKFB2	17.5652	34.1586	0.959524	0.00166463
YIPF7	12.4106	24.1701	0.961647	0.00799923
MCC	9.64256	18.7802	0.961722	0.00709292
GPD1	17.3916	33.9133	0.963462	0.008824
TIMP4	5.80711	11.3456	0.966239	0.00298108
ENSCAFG00000018651	109.603	214.673	0.969847	0.00166463
ENSCAFG00000043069	1.13756	2.24864	0.983106	0.044199
NR1D2	9.57216	19.1299	0.998913	0.00618756
TYW3	6.75546	13.6822	1.01817	0.0324006
ENSCAFG00000024433	4.25612	8.62158	1.01841	0.0228621
CARF	1.13277	2.30547	1.02521	0.0370398
ZNF570	2.05853	4.19674	1.02766	0.0216077
ZNF674	2.58525	5.29226	1.03358	0.0379891
ZBTB40	5.55142	11.502	1.05096	0.00166463

ENSCAFG00000045409,MRPS6,SLC5A3	98.2479	203.855	1.05305	0.00166463
AADAC	6.59142	13.6992	1.05543	0.00709292
GPR22	74.4428	155.414	1.06192	0.00618756
ATXN10	50.2689	105.383	1.06791	0.00166463
LONRF1	3.16254	6.68476	1.07979	0.00166463
C21H11orf54	4.6672	9.8694	1.0804	0.0156032
KBTBD13	0.968805	2.05469	1.08464	0.0204062
TP63	0.9828	2.09305	1.09063	0.0416803
ACSL6	1.89043	4.03308	1.09317	0.0052039
ENSCAFG00000008018	4.53067	9.69064	1.09687	0.0127236
GDA	2.72944	5.92324	1.11778	0.00166463
ENSCAFG00000022744	3855.12	8370.34	1.11851	0.0411794
MOGAT1	10.6439	23.4869	1.14182	0.00166463
GAS1	5.93897	13.2043	1.15272	0.00166463
FASTKD1	1.68129	3.7495	1.15713	0.00414075
PLN	880.215	1963.68	1.15763	0.00166463
RASEF	0.626519	1.40541	1.16556	0.0186537
PHACTR3	0.955746	2.17142	1.18394	0.0135044
SLC4A8	0.902941	2.05463	1.18618	0.00970753
FHIT	6.64879	15.2041	1.1933	0.0111445
PIWIL2	0.573197	1.31519	1.19816	0.026814
cfa-mir-30b	2.39442	5.49827	1.1993	0.0163086
GPCPD1	11.8047	27.1773	1.20304	0.00166463
SLC25A21	9.30615	21.5175	1.20925	0.00414075
ENSCAFG00000013883	8.69563	20.2332	1.21836	0.00166463
ANKRD34B	2.6795	6.27178	1.22691	0.00799923
LAMB4	0.719382	1.68731	1.2299	0.00709292
SLC22A16	0.616617	1.45358	1.23717	0.0370398
ENSCAFG00000017236	17.7923	42.2389	1.24732	0.00166463
ENSCAFG00000022724	18716.3	44671.4	1.25506	0.00166463
FHL5	0.774011	1.85232	1.2589	0.0156032
ABCC5	14.896	35.7953	1.26485	0.00166463
DZANK1	2.15721	5.22236	1.27553	0.0425903
ENSCAFG00000007094	14.0738	34.6548	1.30004	0.00166463
TMEM71	2.06467	5.0924	1.30244	0.00709292
U6	1.76168	4.34544	1.30255	0.00166463
ENSCAFG00000041373	2.22367	5.53401	1.31538	0.00709292
INSIG1	0.621052	1.55334	1.32259	0.0135044
NT5C3A	15.0849	37.8131	1.32579	0.00166463
FMO2	2.23343	5.61144	1.32911	0.00970753
EXPH5	2.3271	5.85298	1.33064	0.00166463
ENSCAFG00000031682	49.8954	127.056	1.34849	0.00166463
PPIG	0.292143	0.745848	1.35221	0.0313973
EYA1	14.4013	37.3691	1.37564	0.00166463
ENSCAFG00000041049	0.908255	2.41023	1.408	0.0251707
TBX18	0.546132	1.45295	1.41166	0.0052039
DOK5	0.71746	1.94053	1.43548	0.0398565
LMO3	16.2153	43.8623	1.43562	0.0186537
LRRC14B	0.507202	1.37261	1.43629	0.0198195
LRRC39	142.488	388.27	1.44622	0.00166463
MSS51	1.27538	3.47665	1.44677	0.0150001
ENSCAFG00000001098	23.2913	64.0488	1.45938	0.00166463
ENSCAFG00000020317	1.28454	3.57098	1.47506	0.0298794

ENSCAFG00000017824	0.675098	1.87755	1.47568	0.0277976
DAO	0.82251	2.29357	1.47949	0.00298108
GRID2	0.303295	0.86237	1.50758	0.0370398
DDIT4L	10.3328	30.8048	1.57593	0.00166463
PFKFB4	2.10066	6.35715	1.59754	0.00166463
Y_RNA	1324.91	4012.82	1.59872	0.0142522
MTTP	0.228201	0.699765	1.61657	0.0277976
ASB12	4.97499	15.3125	1.62194	0.00166463
LRRC9	0.570955	1.76106	1.625	0.00166463
KCNIP2	22.9545	70.9785	1.62861	0.00166463
ENSCAFG00000047853	2.564	7.93016	1.62896	0.00298108
ENSCAFG00000038931	2.41094	7.47983	1.63341	0.00618756
ENSCAFG00000002878	18.9313	58.8408	1.63605	0.00166463
IRS1	1.13725	3.5397	1.63808	0.00166463
SLC38A3	0.338453	1.05499	1.64021	0.0464503
ENSCAFG00000032314	0.500512	1.62412	1.69818	0.0175929
ASB4	1.83347	5.99999	1.71038	0.00166463
TRIQK	1.04103	3.45368	1.73012	0.0298794
RNF128	1.40997	4.92048	1.80313	0.00166463
ENSCAFG00000046556	2.28786	8.08966	1.82208	0.00166463
FGFBP1	2.96384	10.8473	1.8718	0.00166463
ENSCAFG00000023355	0.176412	0.647232	1.87533	0.00166463
ENSCAFG00000046048	0.411136	1.54426	1.90923	0.00414075
ENSCAFG00000041313	0.61054	2.30651	1.91755	0.00166463
KCNA7,NTF4	1.01057	3.93021	1.95944	0.00166463
LGSN	0.679122	2.66661	1.97326	0.00298108
TAF7L	1.15788	4.62139	1.99684	0.00166463
NAP1L2	0.310013	1.41582	2.19124	0.0235189
ENSCAFG00000045934,ENSCAFG00000048914	25.8752	122.247	2.24016	0.00166463
ROS1	1.21018	5.8678	2.27759	0.00166463
GALNT15	0.179936	0.881304	2.29216	0.0163086
ENSCAFG00000025897	1.16461	7.74987	2.73432	0.0454727
DPP10	0.117514	0.859175	2.87012	0.0421371
MLANA	0.377646	3.42637	3.18157	0.0348071
MARCO	0.841976	0	#NAME?	0.00166463
UCN	1.33901	0	#NAME?	0.00414075

*: FPKM.

**: log2(Normal FPKM / CHF FPKM).

Supplementary Table S2. List of genes belonging to GO: 0030198: extracellular matrix organization in DEGs between the LV of CHF and normal dogs.

Gene Symbol	Description
ABL1	ABL proto-oncogene 1, non-receptor tyrosine kinase
ACTN1	actinin alpha 1
ADAM15	ADAM metallopeptidase domain 15
ADAM19	ADAM metallopeptidase domain 19
ADAMTS1	ADAM metallopeptidase with thrombospondin type 1 motif 1
ADAMTS14	ADAM metallopeptidase with thrombospondin type 1 motif 14
ADAMTS2	ADAM metallopeptidase with thrombospondin type 1 motif 2
ADAMTS8	ADAM metallopeptidase with thrombospondin type 1 motif 8
ADAMTS9	ADAM metallopeptidase with thrombospondin type 1 motif 9
ADAMTSL2	ADAMTS like 2
AEBP1	AE binding protein 1
AGRN	agrin
ANTXR1	ANTXR cell adhesion molecule 1
APOA1	apolipoprotein A1
ARF1	ADP ribosylation factor 1
ATXN1L	ataxin 1 like
BGN	biglycan
BMP1	bone morphogenetic protein 1
BMP4	bone morphogenetic protein 4
BSG	basigin (Ok blood group)
CAPG	capping actin protein, gelsolin like
CAPN10	calpain 10
CAPN15	calpain 15
CAPN2	calpain 2
CCDC80	coiled-coil domain containing 80
CCN1	cellular communication network factor 1
CCN2	cellular communication network factor 2
CCN3	cellular communication network factor 3
CD151	CD151 molecule (Raph blood group)
CD34	CD34 molecule
CD44	CD44 molecule (Indian blood group)
COL14A1	collagen type XIV alpha 1 chain
COL15A1	collagen type XV alpha 1 chain
COL16A1	collagen type XVI alpha 1 chain
COL1A1	collagen type I alpha 1 chain
COL1A2	collagen type I alpha 2 chain
COL3A1	collagen type III alpha 1 chain
COL4A1	collagen type IV alpha 1 chain
COL4A2	collagen type IV alpha 2 chain
COL5A1	collagen type V alpha 1 chain
COL5A2	collagen type V alpha 2 chain
COL5A3	collagen type V alpha 3 chain
COL6A1	collagen type VI alpha 1 chain
COL6A3	collagen type VI alpha 3 chain
COL8A1	collagen type VIII alpha 1 chain
COL9A2	collagen type IX alpha 2 chain
CREB3L1	cAMP responsive element binding protein 3 like 1
CRISPLD2	cysteine rich secretory protein LCCL domain containing 2
CTSK	cathepsin K
DPT	dermatopontin

ECM1	extracellular matrix protein 1
EFEMP2	EGF containing fibulin extracellular matrix protein 2
ETS1	ETS proto-oncogene 1, transcription factor
FAP	fibroblast activation protein alpha
FBLN1	fibulin 1
FBLN2	fibulin 2
FBLN5	fibulin 5
FBN1	fibrillin 1
FBN2	fibrillin 2
FMOD	fibromodulin
FN1	fibronectin 1
FNDC1	fibronectin type III domain containing 1
FSCN1	fascin actin-bundling protein 1
FURIN	furin, paired basic amino acid cleaving enzyme
GPIHBP1	glycosylphosphatidylinositol anchored high density lipoprotein binding protein 1
GSN	gelsolin
HAS1	hyaluronan synthase 1
HMCN2	hemicentin 2
HSPG2	heparan sulfate proteoglycan 2
ICAM4	intercellular adhesion molecule 4 (Landsteiner-Wiener blood group)
IGFBP3	insulin like growth factor binding protein 3
IL6	interleukin 6
ITGA3	integrin subunit alpha 3
ITGA5	integrin subunit alpha 5
ITGA7	integrin subunit alpha 7
ITGA9	integrin subunit alpha 9
ITGB2	integrin subunit beta 2
ITGB3	integrin subunit beta 3
ITGB5	integrin subunit beta 5
KERA	keratocan
LAMA2	laminin subunit alpha 2
LAMA4	laminin subunit alpha 4
LAMB4	laminin subunit beta 4
LAMC1	laminin subunit gamma 1
LAMC2	laminin subunit gamma 2
LAMC3	laminin subunit gamma 3
LCP1	lymphocyte cytosolic protein 1
LGI3	leucine rich repeat LGI family member 3
LOXL1	lysyl oxidase like 1
LOXL2	lysyl oxidase like 2
LRP1	LDL receptor related protein 1
LTBP3	latent transforming growth factor beta binding protein 3
LTBP4	latent transforming growth factor beta binding protein 4
MFAP4	microfibril associated protein 4
MGP	matrix Gla protein
MMP14	matrix metallopeptidase 14
MMP2	matrix metallopeptidase 2
MMP23B	matrix metallopeptidase 23B
MMP28	matrix metallopeptidase 28
MMRN2	multimerin 2
MTTP	microsomal triglyceride transfer protein
MXRA5	matrix remodeling associated 5
MYO1E	myosin IE

NCSTN	nicastrin
NID1	nidogen 1
NID2	nidogen 2
NOTCH1	notch receptor 1
NPNT	nephronectin
NTN1	netrin 1
P3H1	prolyl 3-hydroxylase 1
P3H2	prolyl 3-hydroxylase 2
P3H4	prolyl 3-hydroxylase family member 4 (inactive)
P4HA2	prolyl 4-hydroxylase subunit alpha 2
PDGFB	platelet derived growth factor subunit B
PHLDB1	pleckstrin homology like domain family B member 1
PLEC	plectin
PLOD1	procollagen-lysine,2-oxoglutarate 5-dioxygenase 1
PLOD3	procollagen-lysine,2-oxoglutarate 5-dioxygenase 3
PODN	podocan
POSTN	periostin
PRELP	proline and arginine rich end leucine rich repeat protein
PTX3	pentraxin 3
PXDN	peroxidasin
SDC3	syndecan 3
SDC4	syndecan 4
SERPINE1	serpin family E member 1
SERPINH1	serpin family H member 1
SH3PXD2B	SH3 and PX domains 2B
SLIT2	slit guidance ligand 2
SMAD3	SMAD family member 3
SMOC2	SPARC related modular calcium binding 2
SPARC	secreted protein acidic and cysteine rich
SPP1	secreted phosphoprotein 1
SRPX2	sushi repeat containing protein X-linked 2
TGFB1	transforming growth factor beta 1
TGFB2	transforming growth factor beta 2
TGFBI	transforming growth factor beta induced
THBS1	thrombospondin 1
THBS4	thrombospondin 4
TINAGL1	tubulointerstitial nephritis antigen like 1
TNC	tenascin C
TNF	tumor necrosis factor
TNFRSF1B	TNF receptor superfamily member 1B
TNXB	tenascin XB
TSKU	tsukushi, small leucine rich proteoglycan
VCAN	versican
VWA7	von Willebrand factor A domain containing 7
VWCE	von Willebrand factor C and EGF domains
VWF	von Willebrand factor
WT1	WT1 transcription factor
ZDHHC8	zinc finger DHHC-type containing 8

Supplementary Table S3. Hemodynamics, LV function, and hormonal factors in three dog groups.

	Normal	CHF	ACEI
Mean arterial pressure, mmHg	115.4±6.4	90.5±3.8*	92.1±3.1*
LV end-diastolic pressure, mmHg	6.6±0.9	23.4±2.8*	18.8±.2.0*$^{\#}$
Cardiac output, L*min^{-1}	2.91±0.11	1.53±0.10*	2.08±0.11*$^{\#}$
LV end-diastolic diameter, mm	28.6±1.4	39.4±1.6*	35.0±3.1*$^{\#}$
% Fractional shortening	29.7±1.7	8.5±0.8*	17.0±1.6*$^{\#}$
Plasma renin activity, ng*ml^{-1}*hr^{-1}	2.2±0.3	7.4±1.6*	11.6±1.9*
Aldosterone, pg*ml^{-1}	8±2	495±74*	129±52*$^{\#}$
Atrial natriuretic peptide, pg*ml^{-1}	44±8	454±36*	304±28*$^{\#}$

Values are the mean±SEM.

**P*<0.05 vs normal; #*P*<0.05 vs CHF.

Supplementary Table S4. Oligonucleotides used for qRT-PCR

Gene	Forward	Reverse
P3H1	ATGGAGTCCTACTTCCGCCTGG	TCGGCATTCAGGATGCAGTTGTC
P3H2	GAATCGGGTCCCTTCAGGAGTG	CGCTGAGTCCCGTTCAGCTGC
P3H4	TCATGCAGCAGAACCTGGTG	CCAGCTCCATCTCATCATCTGAC
P4HA2	CAATTTGGCCTAAGAAGGGTACAG	CGTTCATGGAACCACTTATTGGAG
PLOD1	TGGGCTCTGGAGGCTTCATCG	GAAGATACGGCAGCGGTGGTC
PLOD3	ACCCGGTCAACCCAGAGAAGC	CACTCCTCTCCCAGGCCCAG
COL1A1	TCCTGGGCCTCAGGGTGCTC	GACCAGCAGGACCAGCATCTC
COL3A1	TGTGCAGTTTGCCCACAGCCTC	CCATTTCTCCCAGGAATACCAG
COL4A1	CCAAAAGGTGACAAGGGTGAC	CATCCCCTGAAATCCAGGTTCAC
hGAPDH	GGGAGCCAAAAGGGTCATCATC	TGGCATGGACTGTGGTCATGAG
cGAPDH	GTCATCATCTCTGCTCCTTCT	CTCAGGGATGACCTTGCCCAC

hGAPDH was used for human samples and *cGAPDH* was used for dog samples. Others was used for human and dog samples.
